# Multiple glycoforms of TrkA interact with N-cadherin during trigeminal ganglion neurodevelopment

**DOI:** 10.1242/jcs.264598

**Published:** 2026-05-11

**Authors:** Caroline A. Halmi, Lisa A. Taneyhill

**Affiliations:** Department of Animal and Avian Sciences, University of Maryland, College Park, MD 20742, USA

**Keywords:** Trigeminal ganglion, N-cadherin, TrkA, Glycosylation

## Abstract

The trigeminal ganglion is a sensory structure derived from neural crest and placode cells, leading to a heterogeneous neuronal population that transmits somatosensory information to the brain. Proper trigeminal ganglion development relies, in part, on neurotrophic signaling, including interactions between nerve growth factor and its receptor, tropomyosin receptor kinase A (TrkA). Although glycosylation is essential for the trafficking of TrkA to the plasma membrane, the glycan profile and functional relevance of these modifications in sensory neurons remains poorly defined *in vivo*. Here, we characterized TrkA glycosylation during chick trigeminal ganglion neurodevelopment and identified multiple glycoforms of TrkA corresponding to partially and fully mature protein states reported *in vitro*. Furthermore, we showed that TrkA interacts with the cell adhesion molecule N-cadherin on neuronal membranes both in cell bodies and axons. The molecular weights of interacting TrkA forms suggest that these associations occur at the plasma membrane as well as on intracellular organelle membranes. Our findings are the first to identify a biochemical association between TrkA and N-cadherin in the trigeminal ganglion, suggesting potential coordination between neurotrophic signaling and cell adhesion during development.

## Teaser

**Summary:** We establish, for the first time, the types of glycans present on TrkA *in vivo* and demonstrate that different TrkA forms interact with N-cadherin within chick trigeminal sensory neurons.

## INTRODUCTION

Cranial ganglia are components of the peripheral nervous system that transmit sensory information from the surrounding environment back to the central nervous system (Schlosser, 2018). The trigeminal ganglion, the largest of all cranial ganglia, extensively innervates the face to relay somatosensory information including pain, touch and temperature (Heimer, 1983). It is derived from two embryonic cell populations, neural crest cells and placode cells, which originate in different anatomical locations but eventually become migratory and coalesce to form this sensory structure (D'Amico-Martel and Noden, 1980; Hamburger, 1961). While both cell types differentiate into neurons, neural crest cells also give rise to supporting glia. This dual embryonic origin of trigeminal sensory neurons leads to a heterogeneous population with distinct molecular profiles and signaling responses (Bhuiyan et al., 2024; Marmigère and Ernfors, 2007; Nguyen et al., 2017).

Among the signaling pathways that are essential for proper trigeminal ganglion development are those initiated downstream of neurotrophins and their cognate receptors, which regulate neuronal survival, outgrowth and subtype specification (Barbacid, 1995; Cohen et al., 1954; Levi-Montalcini and Hamburger, 1953). As development progresses and trigeminal neurons respond to environmental cues, specific subclasses of neurons emerge, including nociceptors, mechanoreceptors and thermoreceptors, which detect pain, mechanical stimuli and temperature, respectively. These neuronal subtypes can be identified in part by the expression of members of the receptor tyrosine kinase (RTK) family known as the tropomyosin receptor kinases (Trks), which are activated by secreted neurotrophic factors (Davies, 1997; Ernfors et al., 1992; Huang et al., 1999a,b; Mu et al., 1993).

TrkA, the high-affinity receptor for nerve growth factor (NGF), is expressed in the majority of nociceptive neurons (Fang et al., 2005). Upon ligand binding and subsequent receptor dimerization, TrkA undergoes autophosphorylation to activate downstream signaling pathways, including MAPK, PI3K/Akt and PLCγ, that promote neuronal survival and differentiation (Huang and Reichardt, 2003; Marlin and Li, 2015). Loss of NGF-TrkA signaling leads to increased apoptosis in small diameter neurons of both the trigeminal ganglion and dorsal root ganglion, confirming its essential role in neuronal survival and health during neurodevelopment (Johnson et al., 1980; Ruit et al., 1992; Smeyne et al., 1994). Although the function of TrkA has been characterized extensively in rat pheochromocytoma cells (PC12) (Negrini et al., 2013; Zhou et al., 1995) and models such as rodent (Fang et al., 2005; McMahon et al., 1994) and chick dorsal root ganglia (Gallo et al., 1997; George et al., 2010; Karlsson et al., 1998; Rifkin et al., 2000; Williams et al., 1995), far less is known about the post-translational mechanisms that regulate TrkA maturation, trafficking and localization to the cell surface, which are crucial for its function as a receptor. Existing information on these processes has been obtained exclusively from studies in PC12 cells, leaving these mechanisms unexplored *in vivo* within cranial sensory ganglia.

Proper localization of TrkA to the plasma membrane relies on a series of regulated processing and maturation steps (Jullien et al., 2002; Martin-Zanca et al., 1989; Watson et al., 1999; Zhou et al., 1995). Data from prior studies indicate that TrkA is synthesized as an immature, unmodified precursor protein. In PC12 cells, the unglycosylated core TrkA protein is approximately 75 kDa, although whether this remains true *in vivo* is unknown (Watson et al., 1999). Next, TrkA undergoes glycosylation in the endoplasmic reticulum (ER) to become a 110 kDa precursor glycoprotein that undergoes further glycosylation in the Golgi to yield a 140 kDa mature form prior to its transit to the cell membrane. Previous studies have shown that the 110 kDa, partially glycosylated form of TrkA is N-linked glycosylated, while the 140 kDa form contains additional sialic residues, likely on these N-linked glycans. Recently, a report has also suggested the presence of O-linked glycosylation on TrkA in cancer cells, although its presence and functional significance, particularly during normal development, are unclear (Lin et al., 2022).

In addition to RTKs, cell adhesion molecules such as cadherins are crucial for neuronal organization and signaling. Cadherins regulate cell–cell adhesion in a calcium-dependent manner and contribute to tissue morphogenesis by influencing proliferation and intracellular signaling (Halbleib and Nelson, 2006). Although multiple cadherins are noted in different tissues, neural cadherin (N-cadherin; Cadherin 2) is broadly expressed in the developing nervous system, including in trigeminal placode cells prior to their delamination, in their neuronal derivatives, and later in all trigeminal sensory neurons irrespective of cellular origin (Hatta and Takeichi, 1986; Shiau et al., 2008). Additionally, N-cadherin knockdown in trigeminal placode cells disrupts initial trigeminal ganglion condensation and later axon outgrowth through cell- and non-cell-autonomous mechanisms involving adhesion (Halmi et al., 2025; Shiau and Bronner-Fraser, 2009). Beyond their adhesive roles, cadherins can also interact with signaling receptors, including RTKs, to functionally regulate downstream signaling, receptor membrane localization and cell behaviors such as migration and proliferation (Andl and Rustgi, 2005; Madarampalli et al., 2019; Nguyen and Mège, 2016; Qian et al., 2004). Dysregulation of these RTK–cadherin interactions are commonly seen in diseases such as cancer, where they contribute to the unchecked proliferation of cells. However, whether such RTK–cadherin interactions occur normally in the developing chick trigeminal ganglion, and how these interactions might influence trafficking and localization of receptors, has not yet been explored.

In this study, we investigate the glycosylation status of TrkA in chick trigeminal sensory neurons, including the relationship between TrkA and N-cadherin. Our results demonstrate that distinct TrkA glycoforms interact with N-cadherin in cell bodies versus axons, and these interactions occur on neuronal cellular membranes, likely on the plasma membrane and the membranes of organelles. Our findings reveal a previously uncharacterized interaction between neurotrophic signaling factor receptors and cell adhesion molecules during sensory ganglia development, raising the possibility that coordination of these pathways may influence TrkA maturation and trafficking.

## RESULTS

### TrkA possesses multiple glycoforms in the trigeminal ganglion

**Fig. 1.**
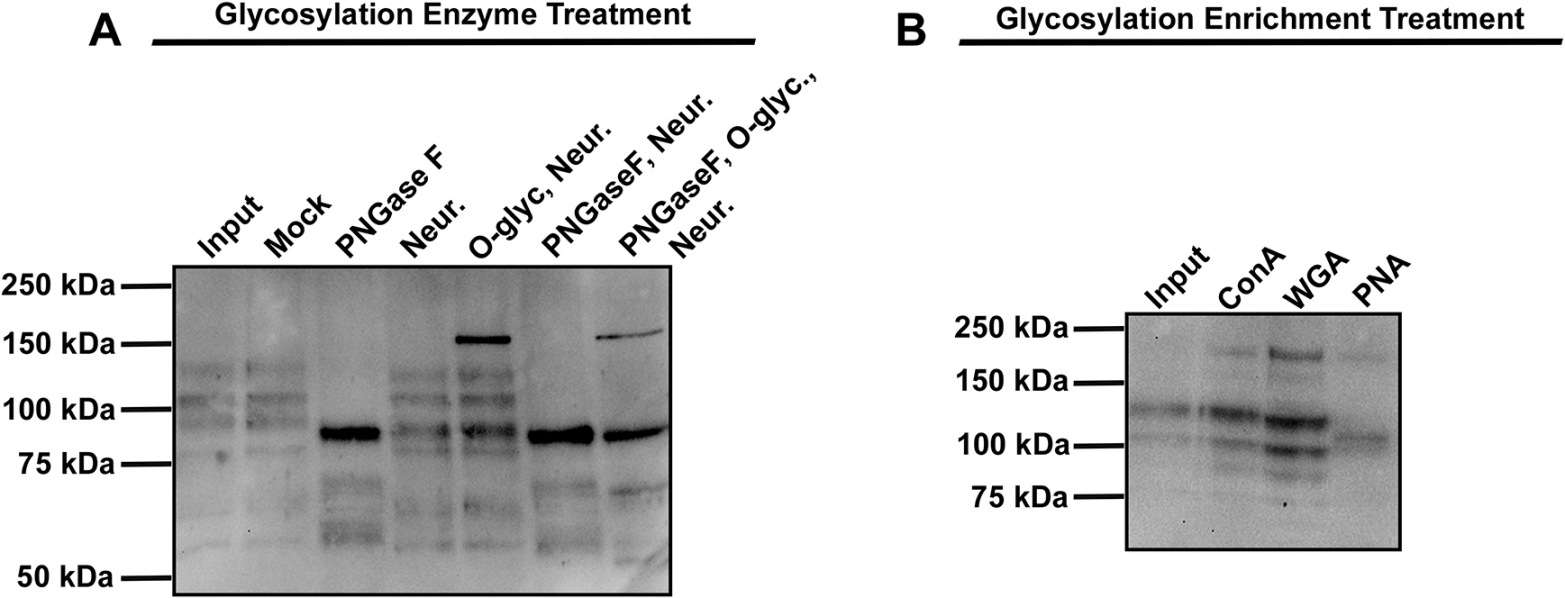
**Different post-translational modifications occur on TrkA.** (A) Pooled E6.5/HH28-30 trigeminal ganglia lysate treated with enzymes to inhibit glycosylation followed by immunoblotting for TrkA (*n*=3 biological replicates). Input: Untreated trigeminal ganglia lysate. Mock (control): Lysate mixed with enzyme buffers. (B) Pooled E6.5/HH28-30 trigeminal ganglia lysate after glycan enrichment followed by immunoblotting for TrkA (*n*=3 biological replicates). Input: untreated trigeminal ganglia lysate. ConA, concanavalin A; Neur., neuraminidase; O-glyc., O-glycosidase; PNA, peanut agglutinin; WGA, wheat germ agglutinin. MSU TrkA antibody was used.

Previous work has demonstrated the glycosylation of TrkA in PC12 cells, which serve as an *in vitro* model of neuronal differentiation. However, TrkA glycosylation has not been studied *in vivo*. Thus, we first sought to establish what types of glycans were present on TrkA in chick trigeminal neurons. We treated embryonic day (E) 6.5/Hamburger Hamilton stage (HH) 28-30 trigeminal ganglia lysate with enzymes that remove specific sugar modifications on proteins (PNGase F: N-linked glycans; O-glycosidase: Core 1 and Core 3 O-linked glycans; neuraminidase: sialic acids) and assessed changes to TrkA banding patterns by immunoblotting (Fig. 1A) with both the input and mock control lanes, the latter containing the enzymatic buffers added to the lysate without any enzymes. Through these enzymatic assays, we observed six bands: the common doublet observed for TrkA in PC12 cells, calculated to be 137 and 107 kDa for our *in vivo* lysate, as well as multiple lower molecular weight bands that were 93 kDa, 76 kDa, 59 kDa and 53 kDa. Notably, in some experiments, we also observed a 223 kDa TrkA band in the input that was reduced in molecular weight in the mock control treatment (Fig. S1).

Upon PNGase F treatment, we observed a total collapse of the 137 kDa, 107 kDa and 93 kDa bands to a singular 88 kDa band, as well as two lower molecular weight bands at 67 kDa and 56 kDa, and persistence of the 53 kDa band. After neuraminidase treatment, we observed a slight shift in the 137 kDa band down to 127 kDa, and a slight reduction in size of the 93 kDa band to 89 kDa, but no change in the 107 kDa, 76 kDa, 59 kDa or 53 kDa bands. Since sialic acid residues are often found at the end of O-glycans and mask the ability of O-glycosidase to cleave these glycans completely, O-glycosidase and neuraminidase treatment were performed together. Interestingly, we observed a prominent 177 kDa band, in addition to the other band shifts observed in the neuraminidase only-treated lysate. When PNGase F and neuraminidase were used together, we observed the same results as the PNGase F only treatment. Lastly, in the combination treatment with PNGase F, O-glycosidase and neuraminidase, we observed the 177 kDa band we had seen previously after combined O-glycosidase and neuraminidase treatment, a prominent band at 88 kDa, which we observed after PNGase F treatment, and two lower bands that were 63 kDa and 52 kDa in size (Fig. 1A, Table 1). We also examined N-cadherin, banding patterns of which only changed in the presence of PNGase F (Fig. S3). This is in line with other reports indicating that N-cadherin is only modified with N-linked glycans (Guo et al., 2009; Langer et al., 2012). Altogether, these enzyme treatments confirm that chick TrkA possesses multiple different types of sugars, including N-linked and sialic acid modifications, and indicate that the 107 kDa TrkA band contains N-linked glycans while the 137 kDa band is both N-linked and sialylated. Our results also indicate the possible occurrence of O-linked glycosylation due to the presence of a 177 kDa band after combined O-glycosidase and neuraminidase treatment.

**Table 1.** Summary of TrkA band shifts and interpretations

Treatment	TrkA bands observed (kDa)	Band shifts/enrichments	Conclusion
Input/mock	223*, 137, 107, 93, 76, 59, 53	Baseline TrkA banding pattern	Multiple forms of TrkA exist in trigeminal ganglia
PNGase F	**88**, **67**, **56**, 53	Collapse of 137, 107 and 93 bands to 88 band	N-linked glycans exist on 137, 107 and 93 bands
Neuraminidase	**127**, 107, **89**, 76, 59, 53	Collapse of 137 band to 127 band and 93 band to 89 band	Sialic acids exist on 137 and 93 bands
O-glycosidase+neuraminidase	**177**, **127**, 107, **89**, 76, 59, 53	177 band appears	O-linked glycosylation detected
PNGase F+neuraminidase	**88**, **67**, **56**, 53	Same as PNGase F	Sialic acid residues are built upon N-linked glycans
PNGase F+O-glycosidase+neuraminidase	**177**, **88**, **63**, **52**	63 and 52 bands appear	Possible unmodified forms of TrkA

Bands in bold denote changed TrkA forms after treatments.

*TrkA band observed variably.

To verify the specificity of our TrkA antibody and the protein bands it identified, we performed two negative control experiments. First, the same immunoblotting experiment was run on enzymatic-treated lysates without the TrkA primary antibody and only the secondary antibody. Here, no bands were detected on the blot, indicating that all bands observed in Fig. 1A are specific and immunoreactive against the TrkA antibody (Fig. S2A). Second, we performed immunoblotting for TrkA on lysates prepared from two negative control tissues (E1.75/HH12-13 heart and somites/lateral plate mesoderm; Cotrina et al., 2000), E6.5/HH28-30 trigeminal ganglia (positive control) and E1.75/HH12-13 forming trigeminal ganglia containing migratory neural crest cells and newly differentiating placodal neuroblasts. We chose the latter tissue so we could examine TrkA in immature neurons/neuroblasts, as RNA-sequencing data from our lab indicates *TrkA* transcripts in that stage group (Leonard et al., 2025). However, the presence, and possible band patterning, of TrkA protein is not known in the newly forming trigeminal ganglion, and we speculated that the banding pattern might look different from that observed in more mature neurons at E6.5/HH28-30. Interestingly, while we only detected the 137 and 107 kDa bands at E6.5/HH28-30, we did note three higher molecular weight bands calculated to be 326, 245 and 161 kDa (Fig. S2B) in all three E1.75/HH12-13 tissues. We also observed a 101 kDa band uniquely expressed in the somite/lateral plate mesoderm sample. Because our secondary only negative control blot also showed no bands, we conclude that these bands are all TrkA immunoreactive (Fig. S2C).

To refine the glycosylation profile of TrkA, we next performed experiments to enrich for different types of glycosylation through the use of concanavalin A (ConA), which binds alpha-linked mannose or terminal glucose residues that are associated with N-linked glycosylation; wheat germ agglutinin (WGA), which recognizes N-acetyl-glucosamine groups or sialic acid; and peanut agglutinin (PNA), which binds galactosyl-(β-1,3)-N-acetyl-galactosamine, a component of the O-glycosylation pathway. Interestingly, TrkA was enriched after all three treatments, further supporting the presence of O-linked glycans on TrkA (Fig. 1B). In addition to the 137 kDa, 107 kDa, 93 kDa and 76 kDa TrkA bands observed in Fig. 1A, ConA and WGA bound glycans on two higher molecular weight TrkA bands at 223 kDa and 177 kDa. PNA also pulled down the 223 kDa band but did not enrich for as many bands as the ConA or WGA treatment. Instead, PNA bound a 117 kDa form of TrkA, along with the 107 kDa TrkA glycoform seen in other treatments (Fig. 1B, Table 2). These experiments confirm that TrkA contains N-linked glycans and sialic acid residues, in line with data from PC12 cells, but also reveal, for the first time, additional O-linked glycosylation in chick sensory neurons. These results suggest that, although similar post-translational modifications occur on TrkA *in vitro* and *in vivo*, additional modifications are also present in chick trigeminal ganglion neurons.

**Table 2.** Summary of TrkA glycan enrichments

Treatment	TrkA bands observed (kDa)	Specificity	Conclusion
ConA	223, 177, 137, 107, 93, 76	α-Mannose and terminal glucose	N-linked glycosylation
WGA	223, 177, 137, 107, 93, 76	GlcNAc and sialic acids	N-linked glycosylation and sialylation
PNA	223, 117, 107	Core O-linked glycan	O-linked glycosylation

GlcNAc, N-acetylglucosamine.

### Inhibiting components of the secretory pathway impacts TrkA processing

To further explore the role of glycan processing of TrkA, we treated trigeminal neuronal explant cultures with validated inhibitors of protein processing and assessed TrkA localization and expression by immunostaining and immunoblotting. Additionally, beta III tubulin (Tubb3) was used as a marker to identify the neuronal cytoskeleton and assist in visualizing neuronal morphology. We hypothesized that each inhibitor would yield a unique change in TrkA expression and/or localization depending upon which portion of the secretory pathway was perturbed. Although we observed multiple TrkA bands, we chose to focus only on the partially and fully glycosylated forms for our analyses, as these are the most prevalent forms that move through the secretory pathway.

**Fig. 2.**
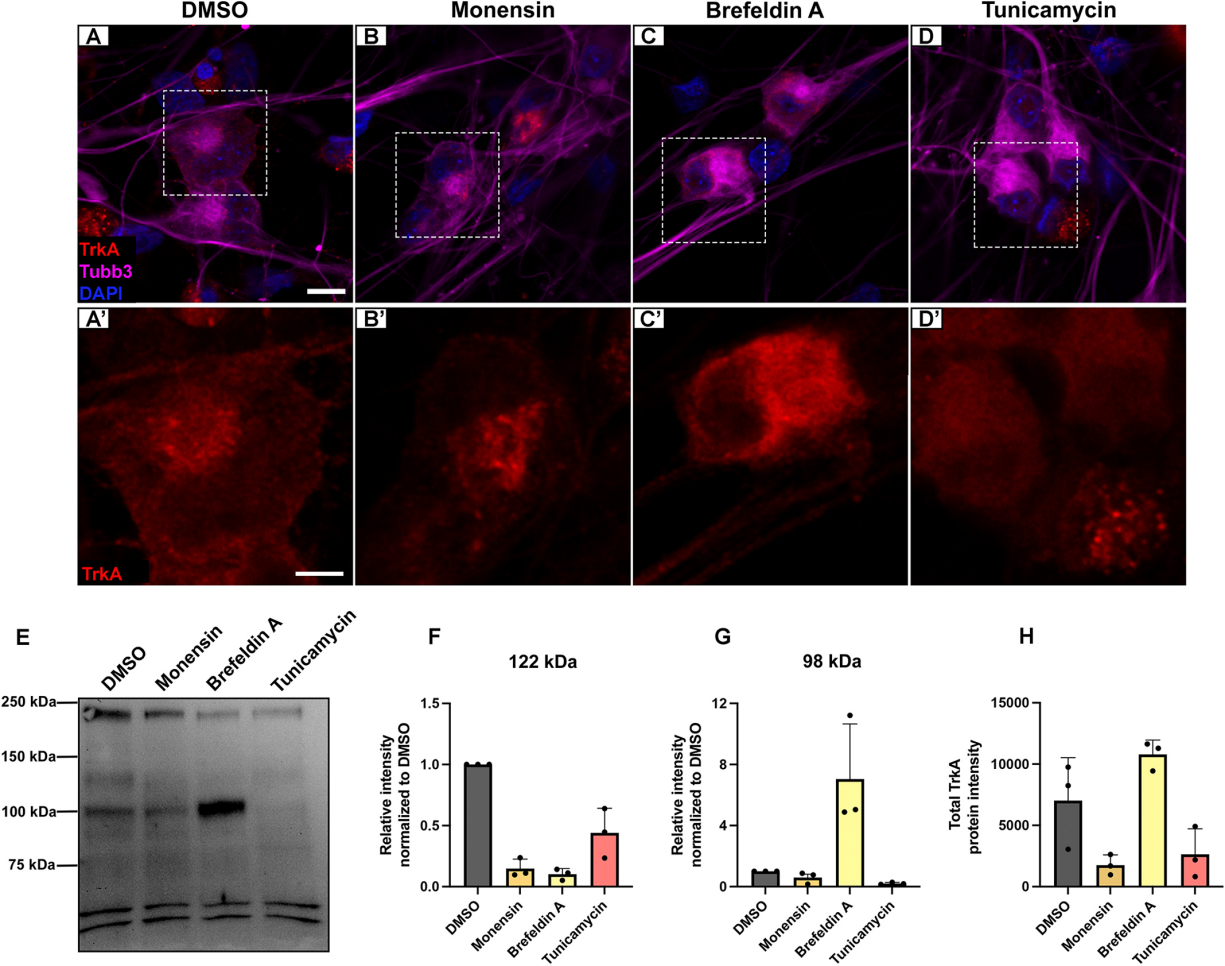
**Inhibitors of the secretory pathway disrupt TrkA expression and glycosylation within trigeminal neurons.** (A-D′) Representative images of cultured trigeminal ganglia following treatment with DMSO (A,A′), monensin (B,B′), brefeldin A (C,C′) or tunicamycin (D,D′), immunocytochemistry for Tubb3 and TrkA, and staining for DAPI (marks nuclei). Scale bars: 10 µm (A, applies to B-D); 5 µm (A′, applies to B′-D′). Airyscan detection was used for all immunocytochemistry images. Dashed boxes indicate the area shown in the single-channel images. (E) Immunoblot for TrkA using lysate prepared from trigeminal ganglia explant cultures following treatment with DMSO, monensin, brefeldin A and tunicamycin. (F,G) Quantification of 122 kDa (F) and 98 kDa (G) TrkA bands normalized against DMSO control. (H) Total TrkA relative protein intensity represented as mean±s.d. (*n*=3 biological replicates). Statistical significance was determined using unpaired *t*-tests with false discovery rate (FDR) control using the two-stage step-up method (Benjamini–Krieger–Yekutieli) and showed no significant differences between treatment groups and DMSO control group. MSU TrkA antibody was used.

In the DMSO control-treated explants, we observed TrkA perinuclearly as well as along the cell membrane (Fig. 2A,A′), and the typical doublet bands (107 kDa and 137 kDa) observed for TrkA that correspond to the partially and fully glycosylated forms (Fig. 2E). We also observed the higher kDa TrkA form in all of our conditions. Interestingly, in these experiments, TrkA ran slightly differently, with the partially and fully glycosylated forms appearing at 98 kDa and 122 kDa, respectively, and the higher form appearing at 216 kDa. Monensin is an antibiotic that inhibits the export of proteins out of the Golgi (Griffiths et al., 1983; Hammerschlag et al., 1982). This treatment yielded an accumulation of TrkA in a perinuclear location, possibly the Golgi given its subcellular distribution and the mechanism of action of monensin (Fig. 2B,B′). Although we observed the typical banding pattern for TrkA by immunoblotting, both the 98 kDa and 122 kDa forms were reduced, with the fully glycosylated form of TrkA being more greatly impacted than the partially glycosylated form (85.2% decrease versus 40.1% decrease; Fig. 2E-G). Brefeldin A is a fungal metabolite that inhibits the anterograde transport of proteins from the ER to the Golgi, causing proteins to accumulate in the ER (Chardin and McCormick, 1999; Reaves and Banting, 1992). After brefeldin A treatment, we observed diffuse expression of TrkA throughout the cytoplasm of neuronal cell bodies (Fig. 2C,C′). This result correlated with the presence of a very prominent 98 kDa TrkA band after immunoblotting (7-fold increase) and a reduction in the intensity of the 122 kDa band (89.8% decrease; Fig. 2E-G). Lastly, tunicamycin is an antibiotic that inhibits N-linked glycosylation of proteins in the ER, leading to an accumulation of unfolded proteins and cellular stress (Struck et al., 1978; Takatsuki et al., 1971). After tunicamycin treatment, we observed a reduction in overall expression of TrkA in the neuronal cultures (Fig. 2D,D′). This phenotype is in line with what we observed by immunoblotting, in that overall TrkA levels appeared reduced (55.7% decrease in the 122 kDa band, 79.5% decrease in the 98 kDa band; Fig. 2E-G). Although TrkA levels fluctuated in response to the different pharmacological agents used, none of our treatments led to a significant reduction in overall TrkA levels (Fig. 2H), suggesting that the concentrations and treatments times of our neuron cultures with these agents did not overtly cause cellular distress.

Even given the results from Fig. 2H, we chose to explore potential impacts to neuronal health and axon outgrowth that might be contributing to the changes in TrkA protein distribution and levels following these treatments. Although axons had grown out in culture after all of our treatments, as indicated by Tubb3 immunostaining, the treated cultures (Fig. S4B-D) appeared qualitatively different to our DMSO cultures (Fig. S4A). Further analyses examining changes to axon outgrowth revealed no significant differences among any of our treatment groups (Fig. S4E), indicating that the pharmacological agents are not inhibiting axon production or outgrowth at this given concentration and incubation time. Given that nuclei can undergo dramatic reductions in size during apoptosis due to fragmentation (Prokhorova et al., 2015), we next examined the size of DAPI-labeled nuclei of TrkA-positive neurons to use as a proxy for overall health. While there were no differences in nuclear size of neurons treated with monensin or brefeldin A compared to our controls, the tunicamycin-treated neurons had significantly reduced nuclear size (Table 3), with 63% of the cells analyzed considered unhealthy. These results suggest that changes to TrkA forms after monensin and brefeldin A treatment are due to disruptions to the secretory pathway, whereas effects from cellular stress and death might be contributing to changes observed after tunicamycin treatment. Taken together, these experiments indicate that blocking the secretory pathway at distinct points alters TrkA distribution and expression, correlating with the portion of the pathway that was perturbed.

**Table 3.** Tunicamycin, but not monensin or brefeldin A, negatively impacts neuronal health

Condition	Unhealthy*/total	Percentage unhealthy	Fisher's exact test against DMSO (*P*)
DMSO	1/11	9%	–
Monensin	1/11	9%	ns
Brefeldin A	3/11	27%	ns
Tunicamycin	8/13	62%	0.0131

ns, not significant.

*Nuclei were considered unhealthy if their area was more than two standard deviations away from the mean area of DMSO nuclei.

### N-cadherin and TrkA show distinct expression in trigeminal neurons

**Fig. 3.**
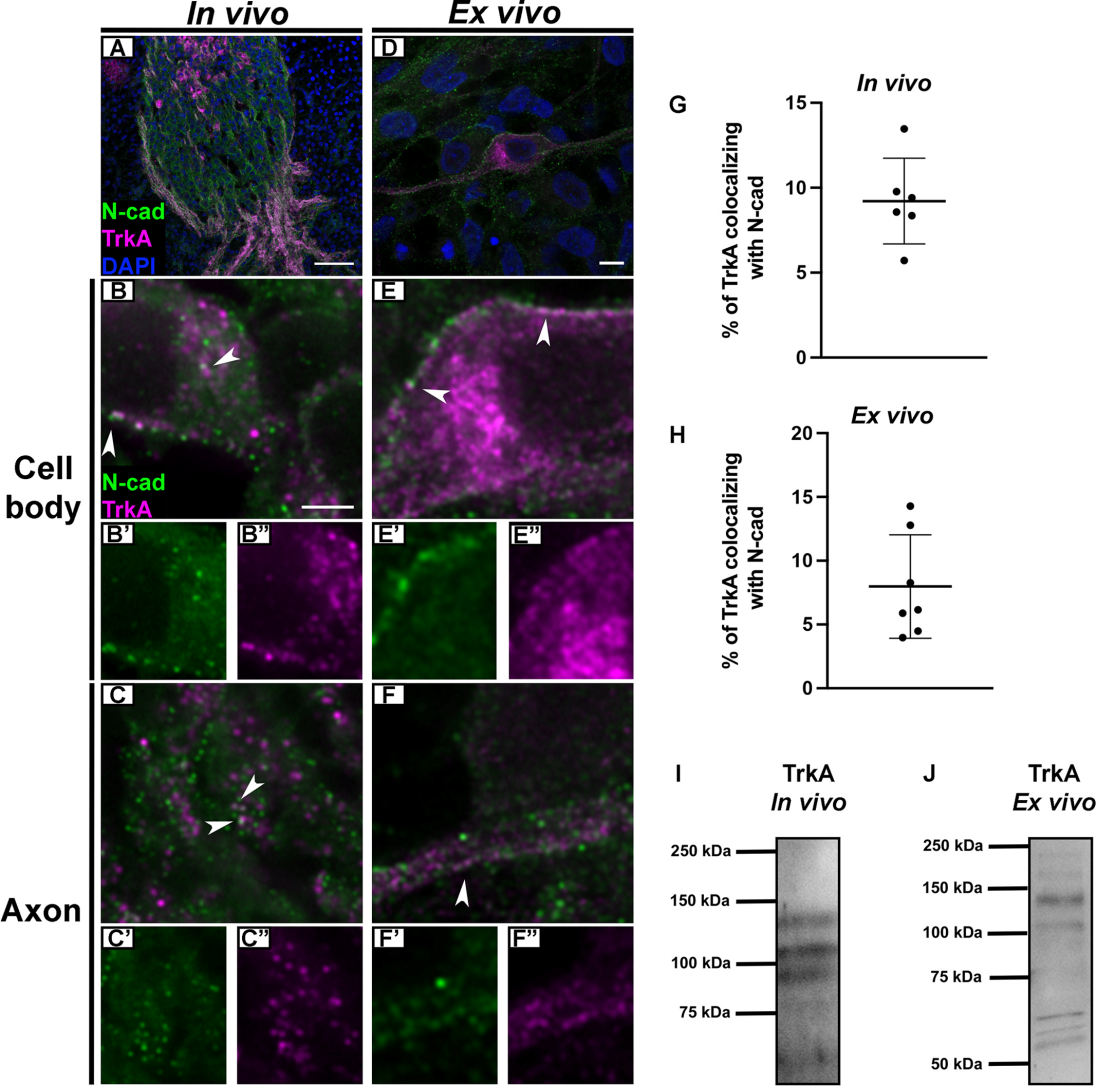
**N-cadherin and TrkA are co-expressed in trigeminal neurons.** (A-F″) Representative immunostaining images for N-cadherin (N-cad) and TrkA of transverse sections through the trigeminal ganglion of an E6.5/HH28 chick embryo (A-C″, *in vivo*) and trigeminal ganglia explant cultures (D-F″, *ex vivo*). (B-B″,E-E″) Higher magnification images for neuronal cell bodies from *in vivo* and *ex vivo* experiments, respectively. (C-C″,F-F″) Higher magnification images for neuronal axons from *in vivo* and *ex vivo* experiments, respectively. Airyscan detection was used for B-C″,E-F″. Scale bars: 50 µm (A); 10 µm (B, applies to B-C″,E-F″; D). Arrowheads indicate colocalization of N-cadherin and TrkA. Neuron in D-F″ is the same as in Fig. S5C-D″. (G,H) Quantification of percentage of TrkA puncta that colocalize with N-cad puncta from the embryo (*in vivo*; *n*=6 biological replicates) (G) and from trigeminal ganglia explant cultures (*ex vivo*; *n*=7 biological replicates) (H). Mean±s.d. (I,J) Immunoblots of pooled E6.5/HH28-30 trigeminal ganglia for TrkA with lysate from the embryo (*in vivo*) (I) and from trigeminal ganglia explant cultures (*ex vivo*) (J). MSU TrkA antibody was used.

Given the known role of cadherin–RTK interactions in other systems (Leckband, 2023), we decided to investigate possible interactions between N-cadherin, known to be expressed in trigeminal sensory neurons (Halmi et al., 2025), and TrkA. To this end, we examined the expression pattern of TrkA, and whether it colocalized with N-cadherin, both in the embryo and in trigeminal ganglion explant cultures. Immunostaining was used to evaluate spatial distribution within trigeminal neurons, and immunoblotting was conducted to assess protein levels and sizes. In the embryo, all trigeminal neurons expressed N-cadherin, while only a subset expressed TrkA (Fig. 3A), as expected (Halmi et al., 2025; Leonard et al., 2022). *In vivo*, we observed colocalization of N-cadherin and TrkA within trigeminal neuron cell bodies, with the two overlapping at both the plasma membrane and intracellularly (Fig. 3B-B″, arrowheads). Colocalization was also detected along axons extending from these neurons (Fig. 3C-C″). In *ex vivo* trigeminal ganglia explant cultures, N-cadherin staining was primarily found at the membrane (Fig. 3D-E′), while the majority of TrkA staining was cytoplasmic (Fig. 3D,E,E″). However, we also observed certain areas within these explants where N-cadherin and TrkA appeared to colocalize at the plasma membrane of neuronal cell bodies and along axons of these explants (Fig. 3E-F″, arrowheads). We next quantified the number of N-cadherin/TrkA double-positive puncta observed in both *in vivo* and *ex vivo* conditions (Fig. S5) and found that 9.2% of TrkA-positive puncta colocalize with N-cadherin-positive puncta *in vivo* (Fig. 3G), compared to 8% *ex vivo* (Fig. 3H).

When examining TrkA by immunoblotting, we observed similar banding patterns and molecular weights *in vivo* (Fig. 3I) and *ex vivo* (Fig. 3J) – 76 kDa, 93 kDa, 107 kDa and 137 kDa – as observed previously (Fig. 1). We also noted two higher molecular weight bands at 223 kDa and 177 kDa in our *ex vivo* lysate, as seen in our glycan enrichment experiments (Fig. 1B). Collectively, these findings indicate that TrkA colocalizes with N-cadherin in trigeminal neurons and maintains similar, but not identical, banding patterns both *in vivo* and *ex vivo*.

### N-cadherin interacts biochemically with TrkA in trigeminal neurons

**Fig. 4.**
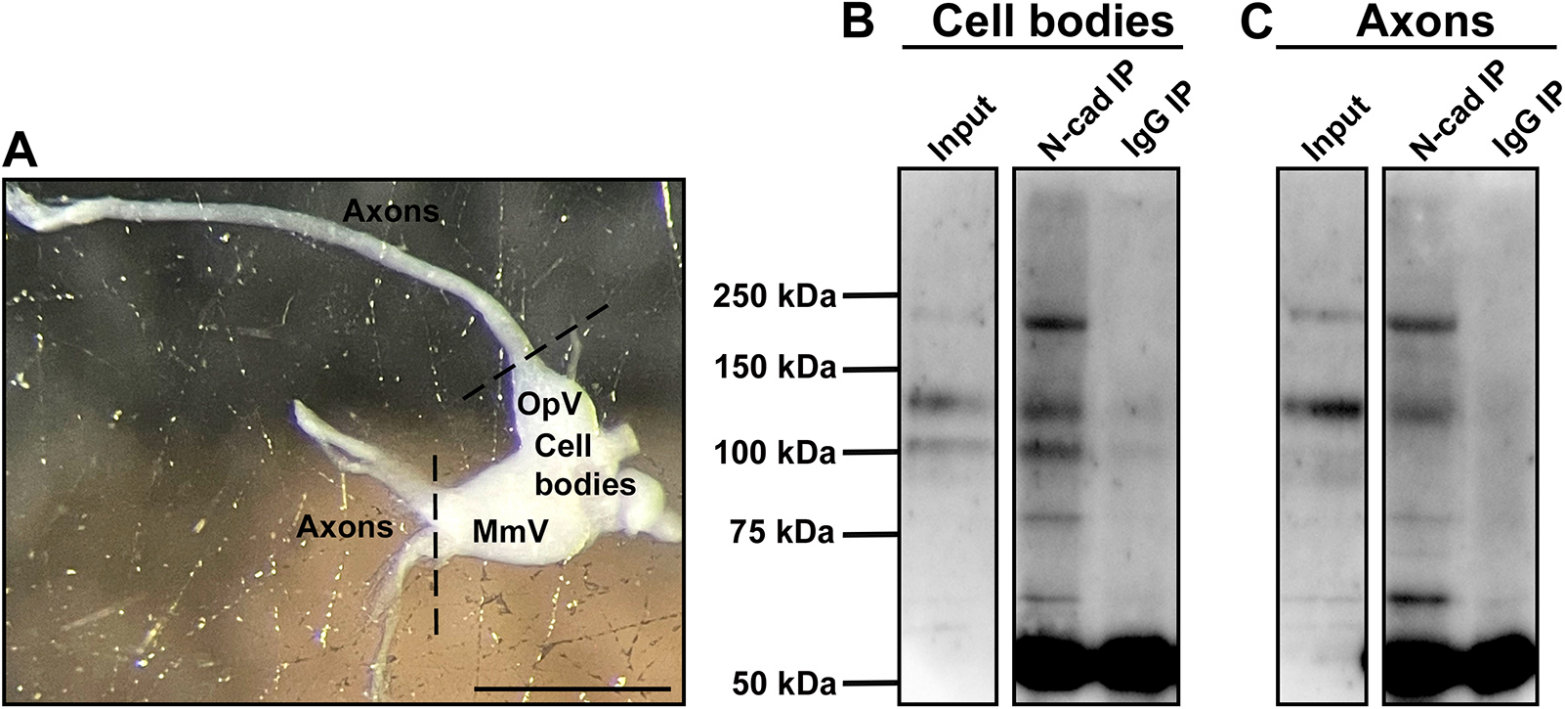
**Different glycosylated forms of TrkA physically interact with N-cadherin in distinct compartments of the trigeminal ganglion.** (A) Dissected trigeminal ganglion from an E6.5/HH28 chick embryo. Dashed lines indicate approximate location where cuts were made to separate trigeminal cell bodies from axons. Scale bar: 1 mm. (B,C) N-cadherin–TrkA co-immunoprecipitation on pooled E6.5/HH28-30 trigeminal cell bodies and axons. Input lanes contain 10% of lysate loaded in immunoprecipitation lanes. IgG, Immunoglobulin G; IP, immunoprecipitation; MmV, maxillomandibular; N-cad, N-cadherin; OpV, ophthalmic. *n*=3 biological replicates. MSU TrkA antibody was used.

Given the colocalization of N-cadherin and TrkA that we observed by immunostaining, we sought to determine whether these two proteins physically interact in the chick trigeminal ganglion. To test this, we used a co-immunoprecipitation assay and examined neuronal cell bodies separately from their axonal projections, as we hypothesized that different forms of TrkA would be expressed in neuronal cell bodies where protein processing occurs versus axons where the mature receptor functions in signaling with NGF. Trigeminal ganglia and their nerve branches were dissected from chick embryos and, once removed, a crude cut was made at the base of the ophthalmic and maxillomandibular lobes to separate trigeminal cell bodies from axons (Fig. 4A, dashed lines). Once lysates were generated for trigeminal cell bodies and axons, an N-cadherin immunoprecipitation with a validated N-cadherin antibody (Halmi et al., 2022) was performed followed by TrkA immunoblotting to detect possible interactions. In the cell bodies experiment, we observed the 223 kDa, 137 kDa and 107 kDa TrkA bands in the input, all of which interact with N-cadherin (Fig. 4B). This differed subtly from what we noted in the axons, where only the 223 kDa and 137 kDa bands were present in the input and after N-cadherin immunoprecipitation (Fig. 4C). Interestingly, in both immunoprecipitations, we also observed two smaller TrkA bands at 75 kDa and 58 kDa. These results align with our hypothesis and indicate that N-cadherin interacts with different forms of TrkA within different compartments of trigeminal neurons.

### TrkA and N-cadherin interact on the membranes of trigeminal neurons

**Fig. 5.**
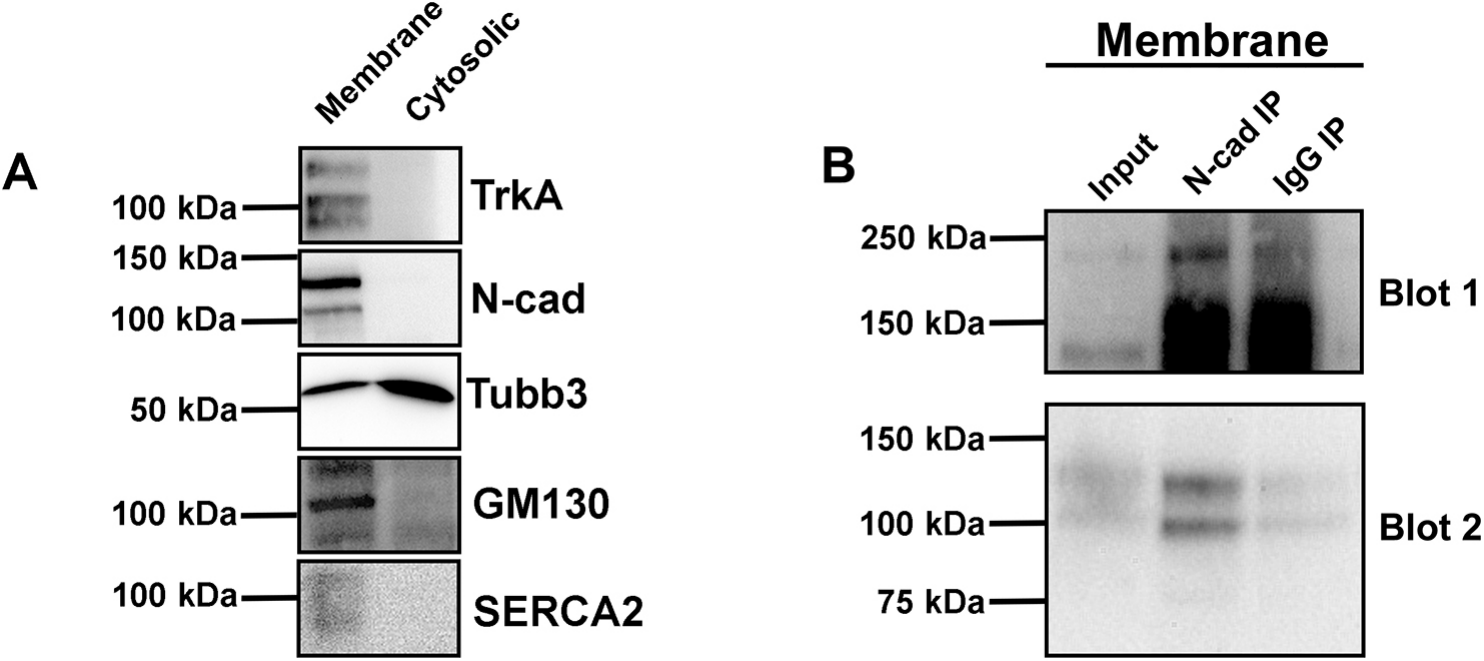
**TrkA and N-cadherin interact on neuronal membranes.** (A) Immunoblots for TrkA, N-cadherin, Tubb3, GM130 (Golgi protein) and SERCA2 (ER protein) conducted on membrane-extracted and cytosolic-extracted protein fractions prepared from pooled E6.5/HH28-30 trigeminal ganglia lysate using the Mem-PER membrane extraction protocol. (B) N-cadherin–TrkA co-immunoprecipitation on membrane-extracted fraction. Input contains 10% of lysate loaded in immunoprecipitation lanes. The top blot image is from one experiment using a high exposure and the bottom blot image is from a separate experiment with a lower exposure. IgG, Immunoglobulin G; IP, immunoprecipitation; N-cad, N-cadherin. *n*=3 biological replicates. Origene TrkA antibody was used.

Our results demonstrate that N-cadherin interacts with the 107 kDa N-linked glycosylated, 137 kDa N-linked and sialylated, and 223 kDa glycoforms of TrkA. However, because only the fully 137 kDa glycosylated form of TrkA is thought to function at the neuronal plasma membrane in signaling with NGF, these results suggest that the interactions we observed between N-cadherin and TrkA are occurring at least partially intracellularly, and potentially in the organelles in which these modifications take place. To explore this further, we separated membrane-bound and membrane-associated proteins from cytosolic proteins in E6.5/HH28-30 trigeminal ganglia. We first verified the accuracy of our extraction method by probing for different proteins and examining in which fraction they were observed. Both N-cadherin and TrkA appeared solely in the membrane-extracted fraction (Fig. 5A). As receptors, these proteins are primarily expressed on the plasma membrane but would also be expected to be embedded in membranes when moving through the secretory pathway. Next, we looked at the cytoskeletal protein Tubb3, which was primarily expressed in the cytosolic fraction, although there was a small amount also found in the membrane fraction. This is not unexpected since Tubb3 interacts with membrane-associated proteins and is found in the mitochondrial membrane of certain cells (Parker et al., 2022; Radwitz et al., 2022). Lastly, we examined Golgi matrix protein 130 (GM130; Golgin A2) and SERCA2, membrane proteins expressed in the Golgi and ER, respectively. Both markers were enriched in the membrane fraction (Fig. 5A). This experiment confirms that our membrane extraction of trigeminal ganglia isolated membrane-bound and membrane-associated proteins from both the plasma membrane and membranes of organelles.

We next performed an N-cadherin immunoprecipitation on the membrane-extracted fraction followed by immunoblotting for TrkA. We observed an interaction between N-cadherin and the 107, 137 and 223 kDa forms of TrkA (Fig. 5B). Given data on the partially glycosylated form of TrkA (107 kDa in our experiments) from PC12 cells as well as the results from our brefeldin A treatment (accumulation of the 107/98 kDa bands after blocking ER to Golgi transport; Fig. 2), these results suggest that the 107 kDa TrkA–N-cadherin interaction is occurring on intracellular membranes. Because sialic residues are added to TrkA in the Golgi, it is possible that the interaction between N-cadherin and the 137 kDa band of TrkA is occurring in the Golgi, at the plasma membrane, or in both locations. This experiment demonstrates that the interactions between N-cadherin and TrkA are occurring on neuronal membranes and are likely happening both intracellularly and at the plasma membrane.

## DISCUSSION

Although post-translational modifications of TrkA have been thoroughly examined in cell lines, whether these same modifications occur *in vivo* remains poorly understood. In this study, we explored the glycosylation of TrkA in chick trigeminal neurons. Our experiments led us to confirm that the 107 kDa and 137 kDa bands commonly associated with TrkA *in vitro* are an N-linked partially glycosylated form and an N-linked and sialylated fully glycosylated form, respectively. Through our experiments, we also observed multiple lower molecular weight forms of TrkA, as well as changes to TrkA after probing for O-linked glycans. Given the co-expression of N-cadherin and TrkA and the presence of cadherin–RTK interactions in other systems, we next examined interactions between TrkA and the cell adhesion molecule N-cadherin and found that N-cadherin binds both partially and fully glycosylated versions of TrkA, as well as a higher and two lower molecular weight forms of TrkA. These interactions were observed in both trigeminal neuron cell bodies and axons, and on neuronal plasma membranes and intracellular membranes, highlighting a previously uncharacterized biochemical association between neurotrophic signaling receptors and cell adhesion molecules in the developing trigeminal ganglion.

### Maturation of chick TrkA involves multiple glycan modifications

To evaluate the identity of the TrkA bands observed in chick trigeminal ganglia, we perturbed or enriched for glycan residues on TrkA. PNGase F treatment led to a single prominent 88 kDa band, demonstrating that the higher molecular weight forms of TrkA are N-linked glycosylated. Additional lower molecular forms persisted after this treatment, suggesting possible intermediate forms of TrkA or proteolytic cleavage fragments. TrkA, along with other RTKs, undergoes proteolytic processing to generate a soluble ectodomain (∼100 kDa) and a membrane-bound fragment (∼41 kDa) (Cabrera et al., 1996; Díaz-Rodríguez et al., 1999). Based on this, it is possible that the 93 kDa band we observed is the soluble ectodomain of TrkA since its retention within the compact trigeminal ganglion and surrounding extracellular matrix is plausible. Intracellular cleavage fragments of TrkA have also been reported in human cells (Merilahti et al., 2017; Merilahti and Elenius, 2019), but none of the TrkA bands observed in our experiments can be intracellular fragments since our TrkA antibody recognizes an epitope in the extracellular domain.

Neuraminidase treatment caused slight molecular weight shifts only to the 137 and 93 kDa bands, identifying these as the only sialylated forms of TrkA. The inability of neuraminidase to collapse the 137 kDa band to 107 kDa suggests that sialylation alone does not account for the size difference, consistent with reports from PC12 cells (Jullien et al., 2002). Other modifications, such as O-linked glycosylation, may contribute, or incomplete enzymatic access to all the sialic acids on TrkA might limit cleavage. Similar incomplete shifts have been reported for TrkA in PC12 cells after neuraminidase treatment (Watson et al., 1999). Combined O-glycosidase and neuraminidase treatment surprisingly yielded a very prominent 177 kDa band. This band likely arises from enzymatic alterations to the 223 kDa TrkA observed in input samples and in N-cadherin-TrkA co-immunoprecipitation. Based on its molecular weight and prior reports (Hartman et al., 1992), this band may represent an NGF–TrkA complex containing Core 1 and 3 O-glycans.

Treatment with PNGase F and neuraminidase together yielded the same result as the PNGase F only treatment, indicating that N-linked glycans form the base to which terminal modifications such as sialic acids are added. Treatment with all three enzymes revealed an additional 52 kDa band that was not found in any other condition. This band may represent a minimally modified or nascent form of TrkA exposed by the enhanced enzymatic accessibility of the combination treatment. However, complete removal of all glycans might be difficult to achieve, as glycosylation often occurs co-translationally (Helenius and Aebi, 2001), and prolonged PNGase F treatment previously failed to detect the ‘core’ TrkA protein (Watson et al., 1999). It is equally plausible that other, non-glycan post-translational modifications may contribute to the observed band heterogeneity such as phosphorylation and ubiquitylation, both of which regulate TrkA function (Georgieva et al., 2011; Yu et al., 2014). Finally, the 223 kDa TrkA form was detected in the input but not the mock control samples, likely due to the presence of additional reducing agents (SDS and DTT) in the latter's denaturation buffer, which, together with Laemmli buffer, may disrupt higher-order TrkA complexes.

To further parse out these different glycans, we performed lectin binding assays. We hypothesized that ConA and WGA would preferentially enrich the 107 kDa N-linked glycosylated and 137 kDa sialylated TrkA forms, respectively. Surprisingly, both bands were enriched with both treatments. Although this contrasts with findings in PC12 cells (Jullien et al., 2002), this discrepancy likely reflects differences between embryonic tissue and cell lines, as TrkA glycosylation *in vivo* may be more complex and heterogeneous. To probe for O-linked glycosylation, we used PNA. Surprisingly, a 117 kDa TrkA band was revealed not observed in other lectin assays or enzyme treatments, suggesting the presence of a low-abundant intermediate TrkA glycoform. Notably, ConA, WGA and PNA all enriched the 177 kDa previously observed following any treatment using O-glycosidase, indicating that this species contains both N- and O-linked glycans. Although this band also persisted after combined PNGase F, neuraminidase and O-glycosidase treatment, it is possible that the enzymatic digestion time was insufficient or that this form of TrkA contains complex secondary structures that make it difficult for PNGase F and neuraminidase on their own to properly access all moieties. Alternatively, this TrkA form may contain Core 2 and Core 4 O-glycans, which are not substrates for O-glycosidase. Definitive classification of these and other TrkA glycoforms with mass spectrometry would be beneficial.

To confirm that the identified bands were not artifactual, we examined TrkA expression in the heart and forming somites, previously considered to lack TrkA and thus serving as negative controls (Cotrina et al., 2000), as well as in forming trigeminal ganglia from the same stage, and E6.5/HH28-30 trigeminal ganglia. More recent transcriptomic studies, however, identified *TrkA* transcripts in these tissues (Anderson et al., 2019; Mok et al., 2024). Importantly, the TrkA forms we detected are consistent with previously reported species (Watson et al., 1999), including NGF–TrkA complexes (Hartman et al., 1992), TrkA multimers (dimers) that are not fully denatured by Laemmli buffer and boiling, or protein aggregates (Shen and Maruyama, 2011). Given the early developmental stage of these tissues, these forms could additionally correspond to highly glycosylated, immature TrkA containing high-mannose N-glycans, as seen for other receptors (Scott et al., 2013; Xiang et al., 2013; Zhen et al., 2003). Treatment with endoglycosidase H, which cleaves high-mannose glycans, would help clarify this.

Interestingly, the 137 and 107 kDa bands observed in E6.5/HH28-30 trigeminal ganglia were absent from all E1.75/HH12-13 tissues that exhibited TrkA immunoreactive bands, suggesting that these bands may represent more-mature neuronal forms within the trigeminal ganglion. Antibody specificity was further supported with a secondary antibody only control that did not detect any of the reported bands, and BLAST analysis confirming epitope specificity for chick TrkA. The closest related sequence, NT-3 growth factor receptor (TrkC), shared only 39% similarity with the TrkA antibody epitope, which is not enough to permit cross-reactivity. Collectively, our investigations support the idea that these bands are developmentally regulated TrkA forms.

### TrkA maturation involves distinct glycosylation events within the secretory pathway

To further define how TrkA glycosylation is regulated during maturation, we used pharmacological agents to perturb the secretory pathway in trigeminal neuron cultures. Prior to these experiments, we optimized inhibitor concentrations and treatment times by evaluating TrkA protein levels and neuronal morphology, selecting conditions that yielded no overt changes to either. Although the observed TrkA molecular weights (98 and 122 kDa) differed slightly from those seen *in vivo* and our other *ex vivo* experiments (107 and 137 kDa), we believe they represent the same TrkA glycoforms and are just disparities due to the nature of SDS-PAGE given the results of our negative control immunoblotting experiments.

Disruption of Golgi export with monensin caused pronounced perinuclear accumulation of TrkA and a reduction in the fully glycosylated form, supporting the conclusion that terminal sialylation occurs in the Golgi (Griffiths et al., 1983; Hammerschlag et al., 1982; Zhu et al., 2024). In contrast, brefeldin A, which blocks ER-to-Golgi transport and causes disruption of the Golgi complex (Chardin and McCormick, 1999; Reaves and Banting, 1992), led to diffuse TrkA staining throughout the soma and enrichment of the 107 kDa band, indicating that this partially glycosylated form is in the ER and further confirming that sialylation occurs largely within the Golgi. Similar perturbed staining patterns have been reported in other types of neurons following brefeldin A treatment (Jareb and Banker, 1997). Lastly, addition of tunicamycin, which prevents all N-linked glycosylation, led to diffuse cytoplasmic TrkA accumulation, marked reductions to the 107 and 137 kDa bands, and the appearance of a distinct 80 kDa form. Because tunicamycin can induce ER stress and protein misfolding (Heifetz et al., 1979), the dim, punctate TrkA staining suggests unhealthy cells and protein processing. The unique detection of the 80 kDa band in this condition supports the notion that this could be an ‘immature’ form of TrkA that possesses little to no glycosylation. Persistence of higher molecular weight bands points to either incomplete deglycosylation or insufficient treatment time to fully expose the core protein. One limitation of these experiments, however, is the lack of validated organelle markers compatible with immunostaining of chick tissue (or available staining kits), which would further strengthen our conclusions regarding subcellular localization of TrkA during its maturation and trafficking.

To distinguish between direct effects on TrkA processing and secondary phenotypes from cellular stress, we measured total TrkA protein levels and nuclear area, the latter used as a proxy for overall neuronal health. Although none of the treatments significantly altered total TrkA levels, tunicamycin significantly reduced the nuclear size of TrkA neurons, indicating that this treatment induces cellular distress. Immature neurons are particularly sensitive to tunicamycin-induced ER stress, exhibiting stronger activation of the unfolded protein response and upregulation of apoptotic signaling (Wang et al., 2015). Therefore, we cannot rule out that some of the TrkA phenotypes we observed after this treatment may result from neuronal stress or death, in addition to direct effects on N-linked glycosylation and TrkA processing.

The use of Tubb3 to visualize the neuronal cytoskeleton helped to substantiate conclusions from our maturation and trafficking experiments as well as shed light on general axon outgrowth under different treatment conditions. While brefeldin A- and monensin-treated cultures did appear to have fewer axons, neuron outgrowth was present and not significantly different in any of the treatments. We cannot say with certainty whether these axons grew out during the experiment or were present at the time of culturing as both trigeminal cell bodies and axons were dissected. Performing a similar experiment with the removal of the axons would better address whether the pharmacological inhibitors actively impact axon outgrowth.

### Different glycoforms of TrkA interact with N-cadherin dynamically in trigeminal neurons

We next examined TrkA and N-cadherin co-expression and found that, both *in vivo* and *ex vivo*, the two colocalized along the plasma membrane and intracellularly, likely within vesicles and perinuclear compartments, within trigeminal neurons. Co-immunoprecipitation experiments revealed that N-cadherin interacts with both the partially glycosylated 107 kDa and fully glycosylated 137 kDa forms of TrkA in cell bodies, but only the 137 kDa form in axons. These findings are consistent with the established model in which TrkA is synthesized and undergoes progressive glycosylation in the cell body, with only the fully mature receptor undergoing anterograde trafficking to axons. Thus, the absence of the 107 kDa band from axonal inputs and immunoprecipitates suggests that this form is a partially glycosylated, intracellular TrkA species.

In contrast, interactions between N-cadherin and the mature 137 kDa TrkA in the cell bodies likely occurs at the plasma membrane or within vesicles undergoing anterograde or retrograde transport. Consistent with this interpretation, studies in rodent sympathetic ganglia demonstrate that mature TrkA can be endocytosed from the surface of cell bodies and transcytosed to axons (Ascaño et al., 2009; Connor et al., 2023; Yamashita et al., 2017), a process that also might occur in chick sensory neurons. In both cell body and axon immunoprecipitations, we observed a higher molecular weight band at 223 kDa, as well as two smaller 76 and 59 kDa bands. As discussed previously, the 223 kDa TrkA form may represent a NGF–TrkA protein complex (Hartman et al., 1992), while the smaller bands could be distinct TrkA glycoforms interacting with N-cadherin during receptor maturation. Interestingly, while this 223 kDa band was observed in both *in vivo* and *ex vivo* experiments, its detection was not consistent. In all experiments, trigeminal ganglia from stages E6.5/HH28-30 were pooled. Therefore, it is possible that this band is predominantly present at a specific developmental stage, and that variation in the relative contribution of embryos from these different stages across experiments underlie its inconsistent detection.

While these data confirm an interaction between N-cadherin and multiple TrkA forms, the nature of this interaction remains unanswered. Cadherin–RTK interactions contribute to cellular processes in both development and disease, often involving direct extracellular binding, as noted for E-cadherin (Cadherin 1)–EGFR and N-cadherin–FGFR complexes (Andl and Rustgi, 2005; Nguyen and Mège, 2016; Qian et al., 2004). In some systems, cadherin binding restricts ligand access to RTKs (Qian et al., 2004; Blaschuk, 2025), although this is unlikely given the presence of a putative NGF-containing 223 kDa TrkA complex. Conversely, N-cadherin may stabilize TrkA at the plasma membrane, as shown for FGFR, where N-cadherin prevents receptor internalization and prolongs signaling (Suyama et al., 2002). Finally, N-cadherin and TrkA may associate through adaptor proteins such as Shc or β-catenin, both of which bind to the cadherin C terminus (David et al., 2008; Xu et al., 1997). Future studies will be necessary to determine whether the interaction between N-cadherin and TrkA is direct or mediated through other proteins.

We lastly sought to explore where the interactions between N-cadherin and TrkA were occurring within trigeminal neurons and found that both the partially (107 kDa) and fully (137 kDa) glycosylated forms of TrkA, as well as the 223 kDa form, co-immunoprecipitated with N-cadherin in the membrane fraction. Given the established role of cadherin–RTK interactions in regulating receptor signaling at the plasma membrane, association of N-cadherin with mature TrkA is consistent with interactions occurring at the cell membrane or within membrane-bound compartments such as signaling endosomes (Harrington and Ginty, 2013; Howe et al., 2001; Sharma et al., 2010). If the 223 kDa band truly represents an NGF–TrkA complex, its association with N-cadherin would likely be at the plasma membrane or within internalized vesicles. Strikingly, N-cadherin also associated with the 107 kDa partially glycosylated TrkA form. Because this immature form has not yet reached the plasma membrane, this interaction likely occurs intracellularly, either on a membrane of the ER or Golgi or within a transport vesicle. Conversely, we cannot rule out that the interaction between the fully mature 137 kDa form of TrkA and N-cadherin solely occurs on the plasma membrane, as it could also be taking place on the Golgi membrane following terminal modifications, or on a vesicle traveling to the cell surface. Although intracellular cadherin–RTK interactions are poorly characterized, ER-to-Golgi trafficking of N-cadherin–FGFR complexes has been reported (Nakamura et al., 2008), raising the possibility that N-cadherin and TrkA similarly associate during transit through the secretory pathway. Future experiments examining the subcellular location of this interaction would give more insight into its functional role.

### Conclusion

In this study, we identified different glycoforms of the neurotrophic receptor TrkA in chick trigeminal sensory neurons and demonstrate that these forms of TrkA interact with N-cadherin on the membranes of neurons. These results contribute to our general understanding of how post-translational modifications and membrane interactions regulate TrkA localization during sensory development. While the functional role of this association has yet to be determined, our findings provide a framework for future studies to assess how N-cadherin may influence TrkA trafficking, signaling and localization in developing neurons. As aberrant RTK and cadherin signaling are increasingly becoming recognized in pathological conditions, and subsequently contribute to altered cell fate and proliferation, understanding how these pathways intersect during normal development could provide important knowledge on how their dysregulation contributes to diseases.

## MATERIALS AND METHODS

### Chick embryos

Fertilized chicken eggs (*Gallus gallus*) were obtained from the Department of Animal and Avian Sciences at the University of Maryland (MD, USA) and incubated at 37°C in humidified incubators on their sides. Embryos were incubated to reach desired HH stages based on times provided therein (Hamburger and Hamilton, 1992). HH stages were used as the primary staging method, while E is reported as an approximate reference.

### Immunohistochemistry

Embryos were collected at desired stages of interest and fixed in 4% paraformaldehyde (PFA) overnight at 4°C with agitation. After fixation, embryos were embedded and sectioned as described previously (Halmi et al., 2025). Briefly, embryos were processed through increasing concentrations of sucrose (5% and 15%) and gelatin (7.5% and 20%), before being embedded in 20% gelatin. Embedded embryos were cryo-sectioned and immunostaining was performed (Halmi et al., 2025). Primary antibodies used were the following: anti-TrkA (a gift from Dr Francis Lefcort, Montana State University, Bozeman, MT, USA; 1:5000; Oakley et al., 1997), anti-N-cadherin (6B3, Developmental Studies Hybridoma Bank; 1:75) and anti-βIII tubulin (ab18207, abcam; 1:500). Secondary antibodies [goat anti-rabbit IgG (A21245, A11037, Thermo Fisher Scientific), goat anti-mouse IgG2a (1080-31, SouthernBiotech) and goat anti-mouse IgG1 (A21121, Thermo Fisher Scientific)] were used at 1:250.

### Trigeminal ganglion explant cultures

Wild-type E6.5/HH28-30 embryos were removed from eggs and stored on ice in neuronal dissection medium (1× Hank's balanced salt solution with 1% penicillin-streptomycin, 10 mM HEPES and 1.4 mM glucose). Trigeminal ganglia were dissected on a SYLGARD-coated dish by first bisecting the embryo and then removing the trigeminal ganglion and axonal projections with tungsten needles. Dissected ganglia were dispersed with a P10 pipette and cultured on two-well glass chamber slides (Thermo Fisher Scientific) coated with poly-L-Lysine (0.01% PLL, Sigma-Aldrich) and fibronectin (0.01%; Sigma-Aldrich). To make the coated slides, PLL was added to each chamber on the glass slides and incubated inside a biosafety cabinet for 30 min at room temperature. The PLL was removed, and slides were washed three times with water. Next, fibronectin diluted in DMEM (Thermo Fisher Scientific) was added to each chamber and incubated at 37°C for a minimum of 2 h. The fibronectin solution was removed, and slides were stored at 4°C until needed (for up to 1 week). Explants were grown in Neurobasal complete media (Thermo Fisher Scientific) supplemented with B27 (1%; Thermo Fisher Scientific), N2 (0.5%; Thermo Fisher Scientific), GlutaMAX (1%; Thermo Fisher Scientific), penicillin/streptomycin (1%; Thermo Fisher Scientific) and with nerve growth factor (Thermo Fisher Scientific), brain-derived neurotrophic factor (Amgen) and neurotrophin-3 (Amgen), all at 50 ng/ml.

### Explant immunocytochemistry

Explant slides were washed once with 1× PBS, followed by fixation in 4% PFA for 30 min at room temperature with agitation. After fixation, slides were rinsed with 1× PBS and stored in 1× PBS at 4°C until ready for immunocytochemistry. A hydrophobic perimeter was drawn around the slides using an ImmEdge Pen (Vector Laboratories) to prevent dehydration of cells, and explants were taken through blocking, primary antibody incubation, and secondary antibody incubation in the absence of coverslips, as described in the ‘Immunohistochemistry’ section. Slides were mounted with DAPI-fluoromount media (SouthernBiotech) and covered with a glass coverslip.

### Confocal imaging

All images were acquired with the LSM Zeiss 800 confocal microscope. Low-magnification images were acquired using a 5× or 20× objective, while high-magnification images were acquired using a 63× oil-immersion objective. High-magnification images were taken using Airyscan detection where indicated. Laser power, gain and offset were kept consistent for the different channels during all experiments where possible. Image processing was performed in Fiji (Schindelin et al., 2012) and Adobe Photoshop CC 2025, and consisted of linear adjustments applied uniformly across images.

### Colocalization analysis

Custom Fiji macros were designed separately for *in vivo* and *ex vivo* image analyses. Rolling-ball background subtraction was applied to each channel to reduce nonspecific signal. For *in vivo* analysis, images were split by channel and thresholded based on intensity to generate binary mask. ‘Analyze particles’ was used to identify individual puncta in each channel. Regions of interest corresponding to puncta from one channel were assessed on the opposing channel to determine spatial overlap. Data were exported as Excel files and analyzed in R to quantify total puncta number and the number of colocalization events between TrkA and N-cadherin. Colocalization was determined as events in which >33% of puncta area overlapped with a puncta in the opposing channel, ensuring confident identification of true colocalization events. The same analytical workflow was applied to *ex vivo* images; however, because of the use of high-magnification images focused on neuronal cell bodies, which did not consistently capture entire axons, neuronal cell bodies were manually outlined prior to puncta detection to restrict analysis to comparable cellular regions. Graphs were generated using Prism GraphPad version 10. Available code contains specific function parameters utilized.

### Immunoprecipitation

Wild-type E6.5/HH28-30 trigeminal ganglia were dissected from embryos and cell bodies were separated from axons using fine tungsten needles. Samples were centrifuged for 5 min at 4°C at 2292 ***g***. Pellets were flash-frozen in liquid nitrogen and stored at −80°C. When needed, samples were lysed in lysis buffer (50 mM Tris pH 7.5, 100 mM NaCl and 0.5% IGEPAL CA-630) supplemented with cOmplete Protease Inhibitor Cocktail (Roche) and 1 mM PMSF for 30 min on ice with periodic gentle vortexing. Lysates were then centrifuged at 20,627 ***g*** for 20 min at 4°C. The soluble protein fraction was then quantified by Bradford assay (Thermo Fisher Scientific). N-cadherin–TrkA coimmunoprecipitations were performed with Protein A/G magnetic beads (Thermo Fisher Scientific) according to the manufacturer's instructions. Briefly, equivalent amounts of protein lysates were incubated overnight with 10 µg of either the N-cadherin antibody (Abcam, ab182) or a rabbit IgG antibody (R&D Systems, AB-105-C) at 4°C with constant mixing. The following day, the lysates were combined with washed magnetic beads (three times in wash buffer) and incubated for 1 h at room temperature with constant mixing. After this, the bound proteins were washed on the beads three times before being eluted off by boiling at 99°C for 7 min in 1× reducing Laemmli sample buffer.

### Immunoblotting

Samples were separated by 8% SDS-PAGE, then transferred to a nitrocellulose membrane (Bio-Rad). The membrane was blocked in 5% milk [in 1× PBS+0.1% Tween-20 (PTW)] for 30 min at room temperature followed by overnight incubation at 4°C with the following primary antibodies diluted in blocking solution: N-cadherin (MNCD2, Developmental Studies Hybridoma Bank; 1:100), TrkA [gift from the laboratory of Dr Frances Lefcort, Montana State University (MSU), 1:5000; TA806413, Origene, 1:1000], β-actin (sc-47778, Santa Cruz Biotechnology; 1:1000), GM130 (sc-55590, Santa Cruz Biotechnology, 1:100), SERCA2 (sc-53010, Santa Cruz Biotechnology, 1:100) and Tubb3 (ab78078, Abcam, 1:500). Membranes were washed three times for 10 min each in PTW before incubation with secondary antibodies [species- and isotype-specific horseradish peroxidase: HRP goat anti-mouse IgG (115-035-003, Jackson Immunology), HRP goat anti-mouse IgG1 (115-035-205, Jackson Immunology), HRP goat anti-rat IgG (112-035-143, Jackson Immunology), goat anti-mouse, HRP mouse IgGk light chain-binding protein (sc-516102, Santa Cruz Biotechnology)] diluted in blocking solution for 1 h at room temperature. Membranes were washed three times for 10 min each in PTW. Antibody detection was performed using Supersignal West Pico or Femto chemiluminescent substrates (Thermo Fisher Scientific) and visualized using a ChemiDoc XRS system (Bio-Rad). For secondary only negative control experiments, blots were processed as indicated above, with the omission of any primary antibody during the overnight incubation at 4°C. See blot transparency figure (Fig. S6) for all uncropped blots.

To calculate protein molecular weights, a straight vertical line was drawn through the protein ladder and a profile plot was generated on Fiji. The pixel value for each peak, which corresponded to a molecular weight marker, was recorded. The log_(10)_ value for each molecular weight marker was calculated and a graph was generated with the migration distance (pixel value) along the *x*-axis and the log_(10)_ value along the *y*-axis. A linear regression was fitted and the corresponding equation of the line was used to calculate the molecular weight of all unknown bands.

### Enzymatic treatments of lysate

For all enzymatic treatments, equal amounts of trigeminal ganglia lysate were used. PNGase F (NEB, P0704S), O-glycosidase (NEB, P0733S) and α2-3,6,8 neuraminidase (NEB, P0720S) were added to lysate per manufacturer's guidelines. Samples were incubated for 2-4 h at 37°C. After incubation, 1× reducing Laemmli sample buffer was added to each sample, and samples were boiled at 99°C for 7 min before protein separation by SDS-PAGE.

### Glycoprotein enrichment

#### ConA and WGA

To enrich for N-linked glycans and sialic acid residues, ConA and WGA Glycoprotein Isolation kits were used, respectively (Thermo Fisher Scientific, 89804, 89805). Briefly, the resin was first washed three times with 1× Wash Buffer. Trigeminal ganglia lysate was added to the washed resin and 1× Wash Buffer was added to reach a final volume of 250 µl. Lysate/resin mixtures were incubated for 1 h at room temperature with constant mixing. After incubation, glycoprotein-bound resin was washed four times with 1× Wash Buffer. After the final wash, 1× reducing Laemmli sample buffer was added to each sample, and samples were boiled at 99°C for 7 min before protein separation by SDS-PAGE. This experiment was performed on unconjugated agarose resin as described above for a control.

#### PNA

To enrich for O-linked glycans, biotinylated PNA (Vector Laboratories, B-1075-5) was used. Prior to enrichment, the lysate was treated with α2-3,6,8 neuraminidase (NEB, P0720S) to remove sialic acid residues. In a microcentrifuge tube, neuraminidase-treated lysate, biotinylated PNA (10 µg/ml) and Wash/Binding Buffer (1× PBS with 0.05% Tween-20) were combined to reach a volume of 500 µl and incubated overnight at 4°C with constant mixing. The next day, streptavidin Sepharose resin (Thermo Fisher Scientific, 20349) was washed with Wash/Binding Buffer three times before adding the lysate/lectin solution to it. Lysate/resin mixtures were incubated for 1 h at room temperature with constant mixing. After incubation, glycoprotein-bound resin was washed four times with Wash/Binding Buffer. After the final wash, 1× reducing Laemmli sample buffer was added to the sample, which was then boiled at 99°C for 7 min before protein separation by SDS-PAGE. This experiment was performed on Sepharose resin without biotinylated PNA as described above for a control.

### Inhibitor treatment of explant cultures

Wild-type E6.5/HH28-30 trigeminal ganglia explant cultures were grown for 1 day *in vitro* as described in the ‘Trigeminal ganglion explant cultures’ section. Tunicamycin (Sigma-Aldrich, T7765), Bbrefeldin A (Sigma-Aldrich, B6542) and monensin (Sigma-Aldrich, M5273) were re-suspended in DMSO to obtain a concentration of 1 mg/ml. The next day, all inhibitors, and the DMSO control, were separately added to Neurobasal complete media with brain-derived neurotrophic factor (Amgen, 50 ng/ml) and neurotrophin-3 (Amgen, 50 ng/ml), at a final concentration of 1 µg/ml, and each inhibitor (or control) was added to the explant cultures for 4 h. After incubation, the media was removed, and explants were washed in 1× PBS before either being fixed in 4% PFA for immunostaining or flash-frozen in liquid nitrogen and stored at −80°C to be processed for immunoblotting.

Axon outgrowth was quantified using high-magnification (63×) images of neuron cultures stained with Tubb3. Cell bodies were excluded from all images to isolate only axonal projections. Tubb3 images were then converted to binary (black-and-white) format, and the total area of axons was measured for each treatment condition. Statistical analyses were run on Prism GraphPad version 10.

To measure cellular health, the area of individual DAPI-labeled nuclei from TrkA/Tubb3-double positive neurons was measured using the polygon selection tool on Fiji for each treatment condition and analysis was run on Prism GraphPad version 10.

### Extraction of membrane and cytosolic protein fractions from trigeminal ganglia

Membrane and cytosolic proteins from E6.5/HH28-30 trigeminal ganglia were isolated using the Mem-PER Plus Membrane Protein Extraction Kit (Thermo Fisher Scientific, 89842), per manufacturer's guidelines. Briefly, flash-frozen trigeminal ganglia were thawed on ice and washed once with Cell Wash Solution. Permeabilization Buffer was added to trigeminal ganglia, mixed until a homogenous solution was obtained, and incubated for 10 min at 4°C with constant mixing. The solution was centrifuged at 16,000 ***g*** for 15 min at 4°C to pellet permeabilized cells. The supernatant, which contained the cytosolic protein fraction, was removed and transferred to a new tube. Solubilization Buffer was then added to the pellet containing the membrane-bound proteins and mixed until a homogenous solution was obtained. The solution was incubated for 30 min at 4°C with constant mixing before being centrifuged at 16,000 ***g*** for 15 min at 4°C. The supernatant containing membrane-associated proteins was transferred to a new tube before both fractions were quantified by Bradford assay for subsequent experimentation.

## Supplementary Material



10.1242/joces.264598_sup1 Supplementary information
