# Supplementary material for: Multiple glycoforms of TrkA interact with N-cadherin during trigeminal ganglion neurodevelopment
Source: J Cell Sci. 2026 May 11;139(9):jcs264598. doi: 10.1242/jcs.264598 (PMC13245897; doi:10.1242/jcs.264598)
Supplement: Supplementary information [file joces-139-264598-s1.pdf]

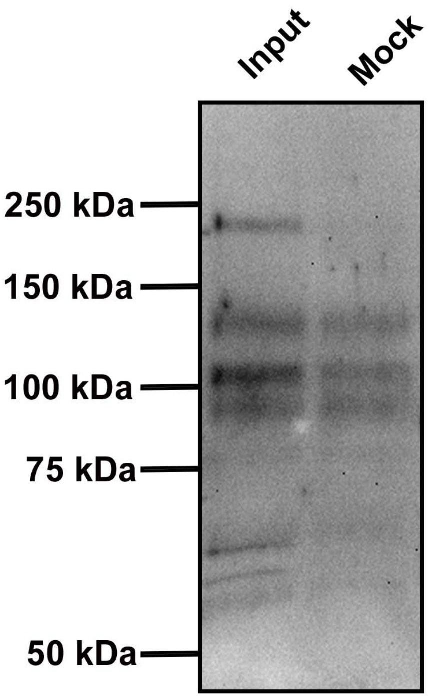

Fig. S1. Higher molecular weight TrkA is observed in trigeminal ganglia lysate. Pooled E6.5/HH28-30 trigeminal ganglia lysate treated with enzymes to inhibit glycosylation followed by immunoblotting for TrkA. Input: Untreated trigeminal ganglia lysate. Mock (control): Lysate mixed with enzyme buffers (N=3). MSU TrkA antibody was used.

Journal of Cell Science • Supplementary information

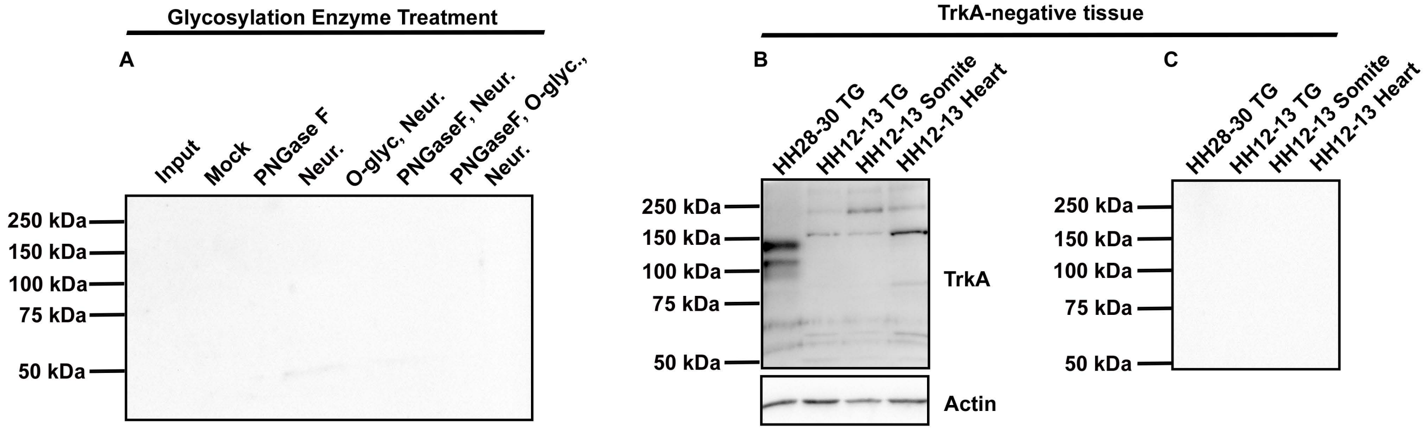

Fig. S2. Negative control immunoblotting experiments for Figure 1. (A) Pooled E6.5/HH28-30 trigeminal ganglia lysate treated with enzymes to inhibit glycosylation followed by immunoblotting with only a rabbit IgG HRP secondary antibody. Input: Untreated trigeminal ganglia lysate. Mock (control): Lysate mixed with enzyme buffers. PNGase F, Neuraminidase, O-glycosidase (O-glyc.). (B) Immunoblotting for E1.75/ HH12-13 embryos (and E6.5/HH28-30 trigeminal ganglia as a positive control) to examine the specificity of the TrkA antibody. MSU TrkA antibody was used. (C) Repeat experiment from (B), without the addition of the TrkA antibody, and immunoblotting with only a rabbit IgG HRP secondary antibody.

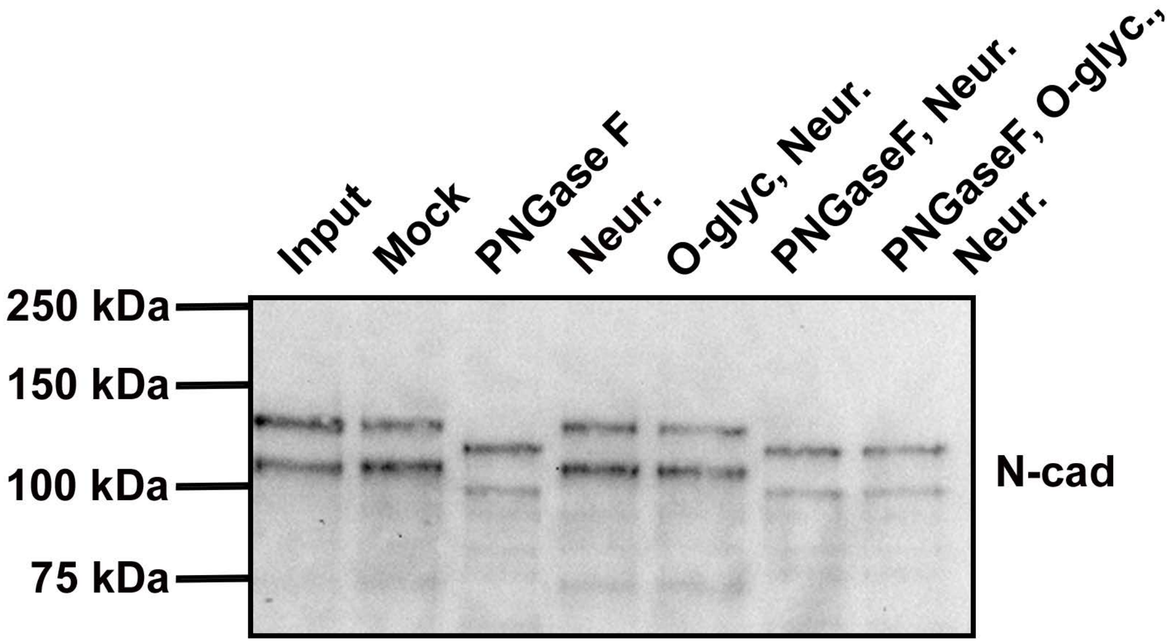

Fig. S3. N-cadherin is N-linked glycosylated. Pooled E6.5/HH28-30 trigeminal ganglia lysate treated with enzymes to inhibit glycosylation followed by immunoblotting for N-cadherin (N-cad). Input: Untreated trigeminal ganglia lysate. Mock (control): Lysate mixed with enzyme buffers. PNGase F, Neuraminidase, O-glycosidase (O-glyc.) (N=2).

Journal of Cell Science • Supplementary information

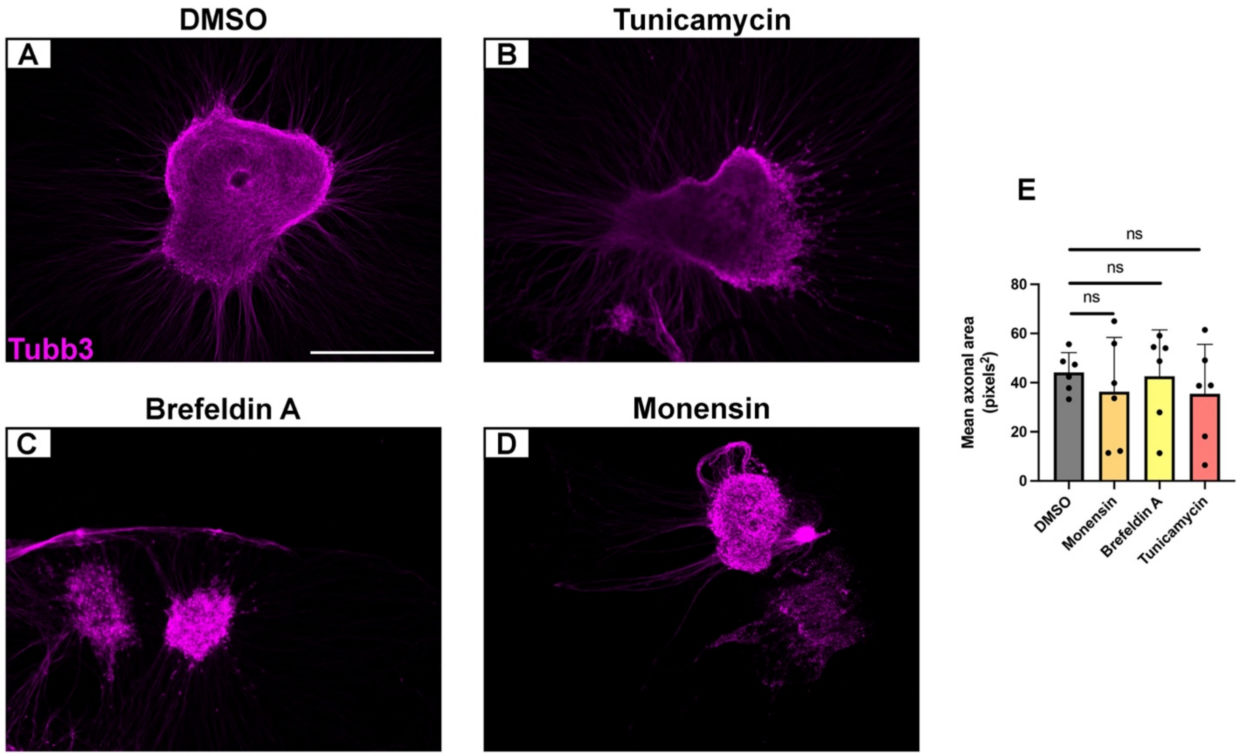

Fig. S4. Trigeminal ganglion neuron cultures retain axon outgrowth after treatments with inhibitors of the secretory pathway. (A-D) Representative images of cultured trigeminal ganglia following treatment with DMSO (A), Tunicamycin (B), Brefeldin A (C), or Monensin (D), and immunocytochemistry for Tubb3. Scale bar in (A) is 500 µm and applies to (B-D). (E) Axonal signal area represented as averages ± SD (N=6). Statistical analysis was determined using unpaired t-tests.

Journal of Cell Science • Supplementary information

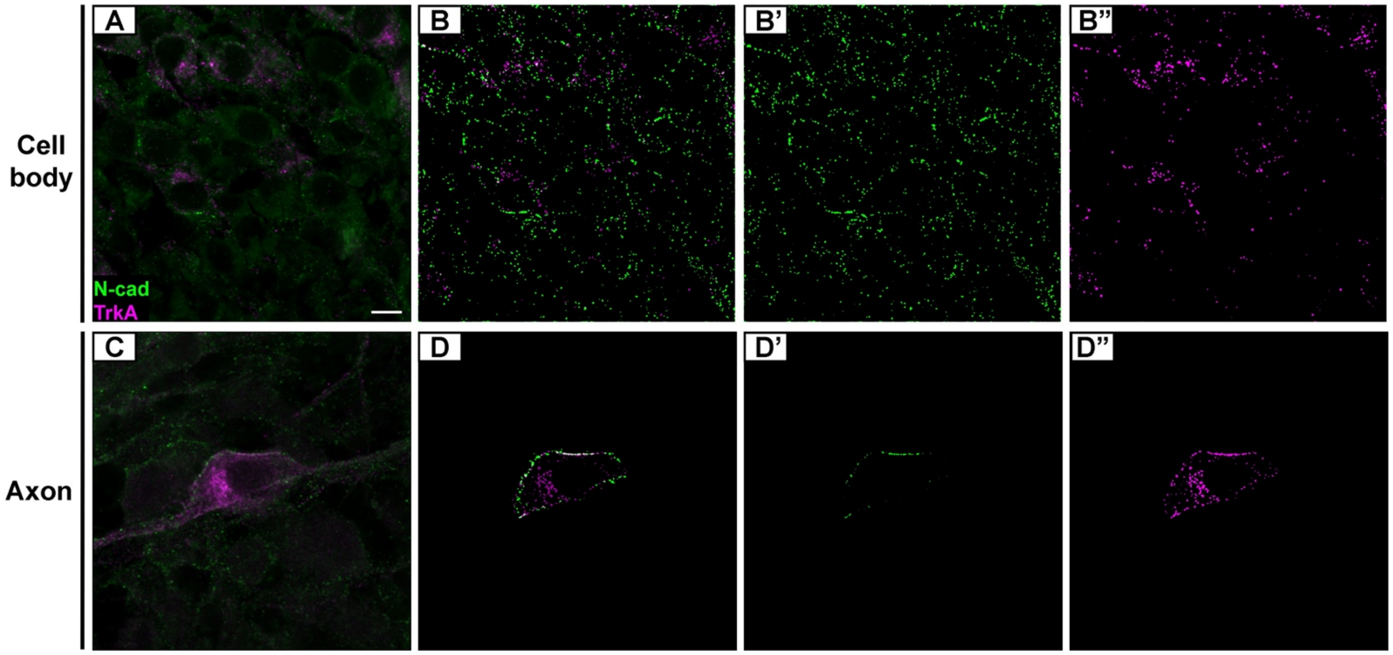

Fig. S5. N-cadherin and TrkA colocalization analysis. Representative images from *in vivo* (A-B") and *ex vivo* (C-D") immunostaining experiments with N-cadherin (green) and TrkA (purple) antibodies. (A, C) Raw microscopy images used for colocalization analysis. (B, D) Masked puncta overlay for N-cadherin and TrkA channels. White puncta denote areas where puncta from both channels overlap. (B', B", D', D") Individual masks for N-cadherin and TrkA, respectively. Scale bar in (A) is 10 µm and applies to all images. Neuron in C-D" is the same as in Fig. 3D-F"

Fig. 1.

A: Merged w/ ladder

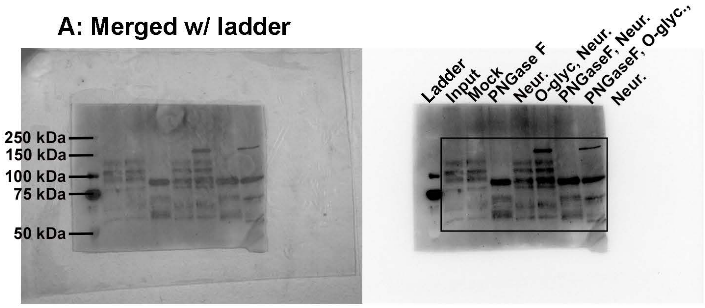

B: Merged w/ ladder

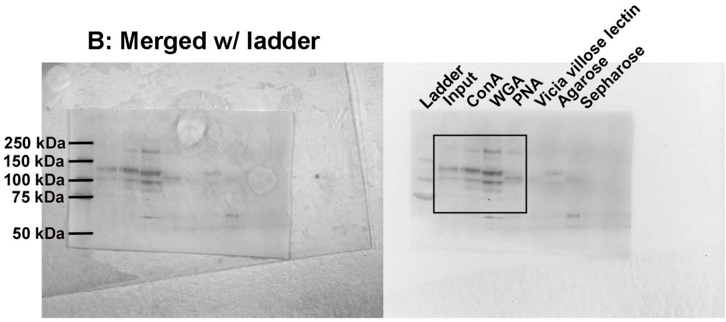

Fig. 2.

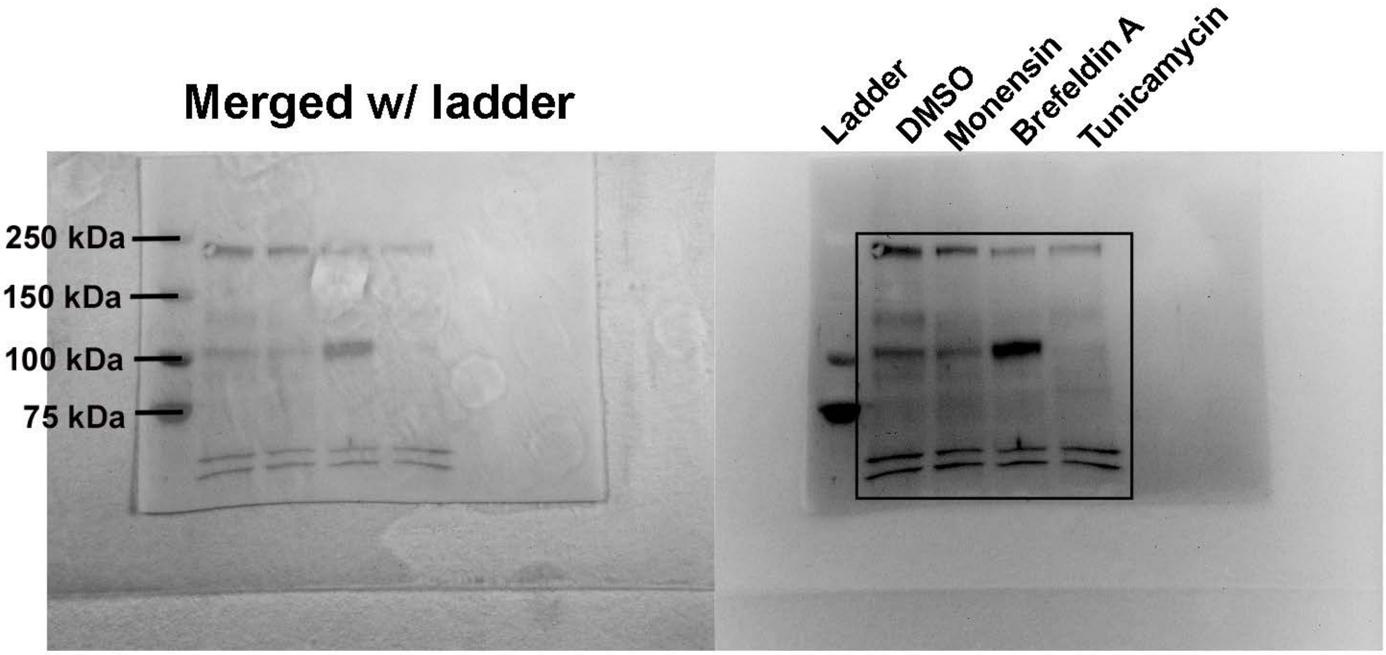

Fig. 3.

I: Merged w/ ladder

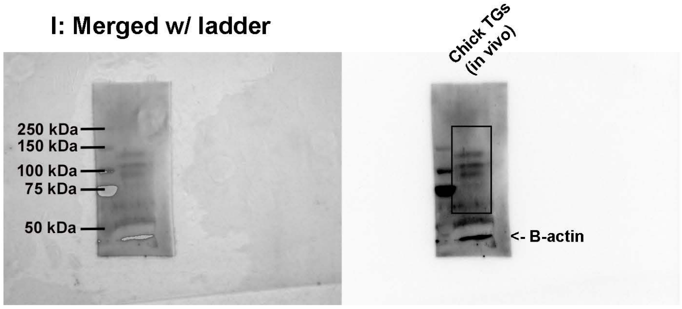

J: Merged w/ ladder

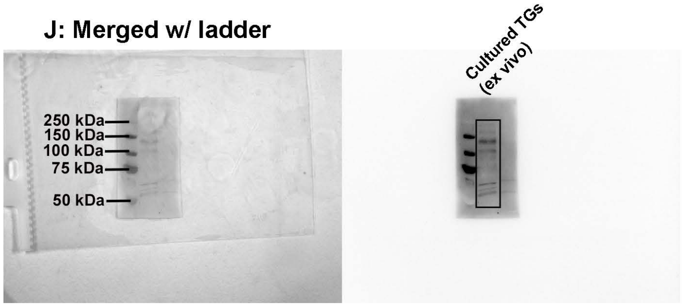

Journal of Cell Science • Supplementary information

Fig. 4.

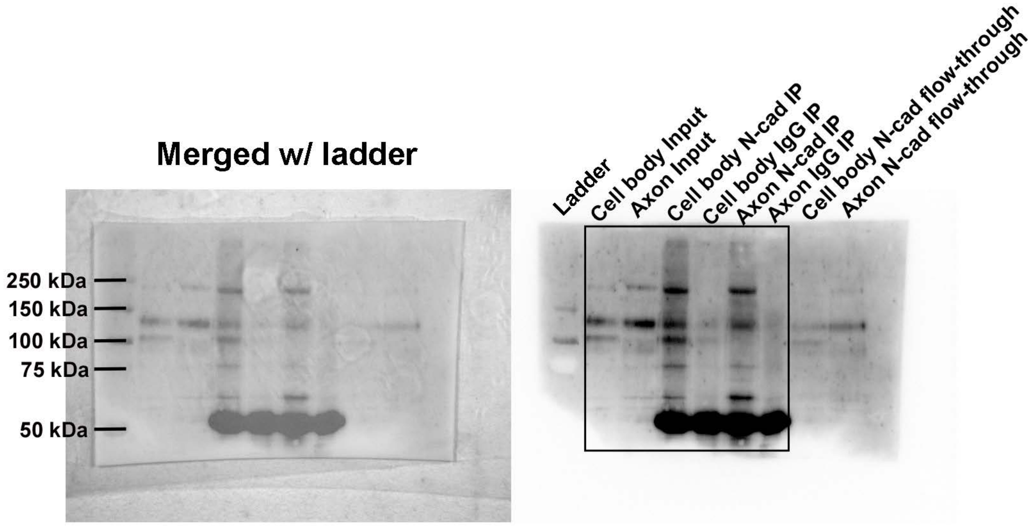
Merged w/ ladder
250 kDa
150 kDa
100 kDa
75 kDa
50 kDa
Ladder
Cell body Input
Axon Input
Cell body N-cad IP
Cell body IgG IP
Axon N-cad IP
Axon IgG IP
Cell body N-cad flow-through
Axon N-cad flow-through

Fig. 5.

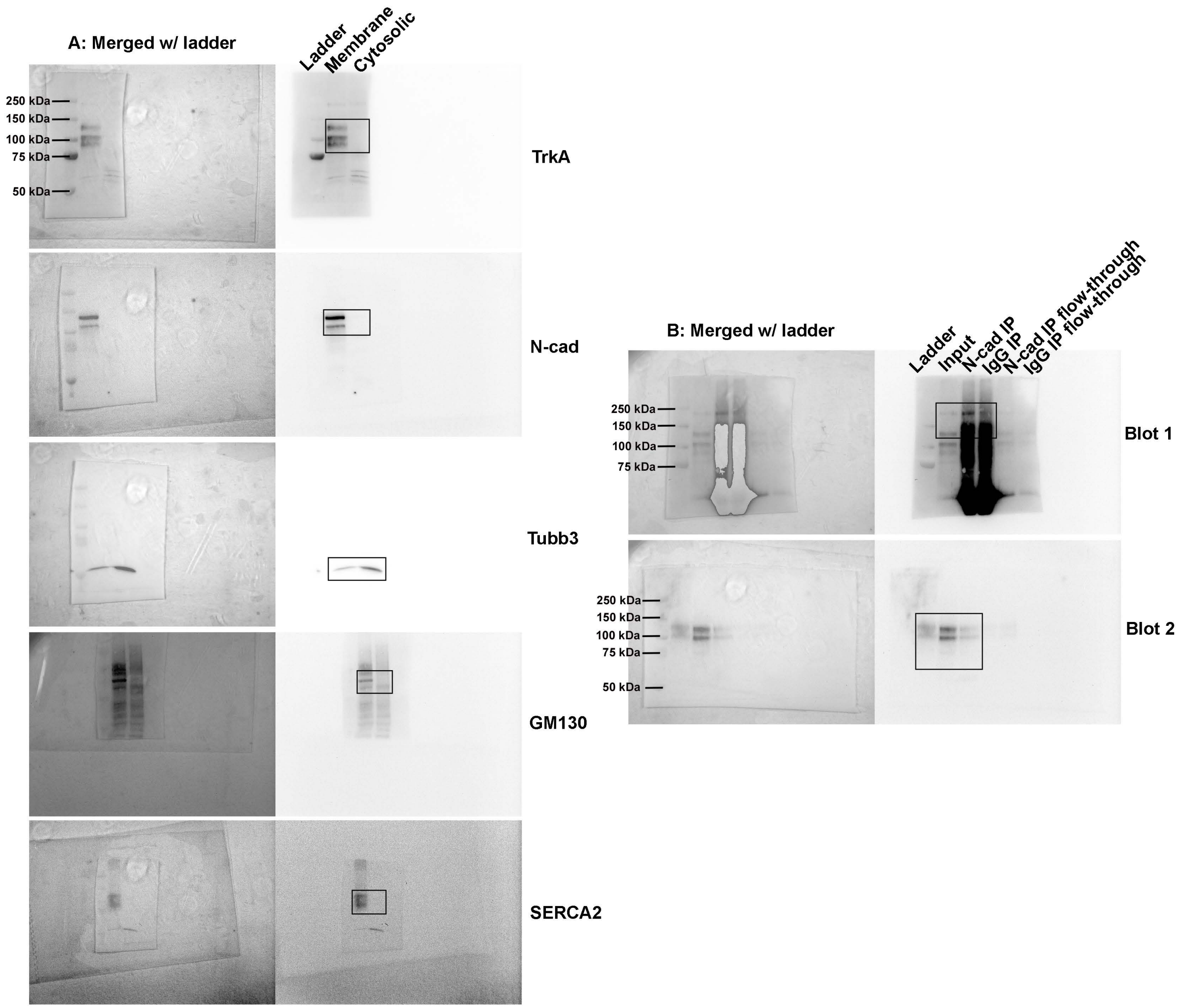
A: Merged w/ ladder
Ladder
Membrane
Cytosolic
250 kDa
150 kDa
100 kDa
75 kDa
50 kDa
TrkA
N-cad
Tubb3
GM130
SERCA2
B: Merged w/ ladder
Ladder
Input
N-cad IP
IgG IP
N-cad IP flow-through
IgG IP flow-through
Blot 1
Blot 2

Fig. S1.

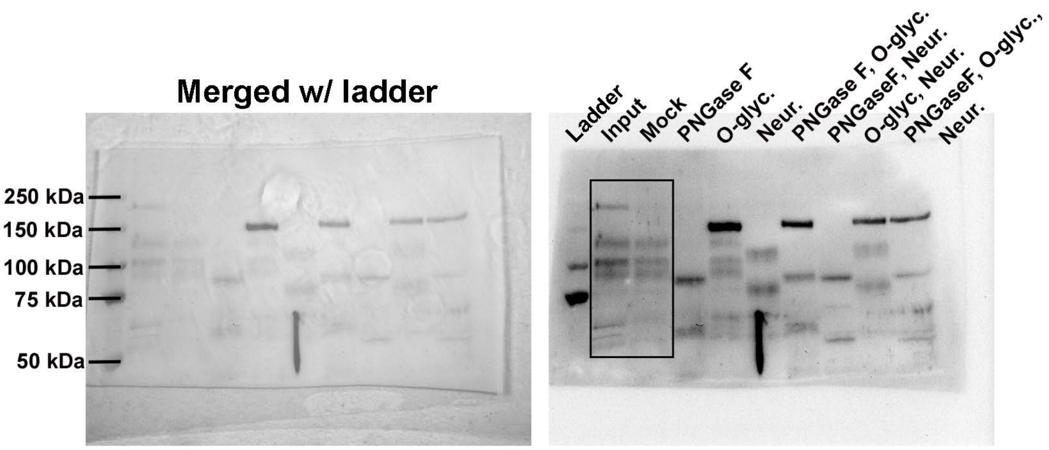

Fig. S2.

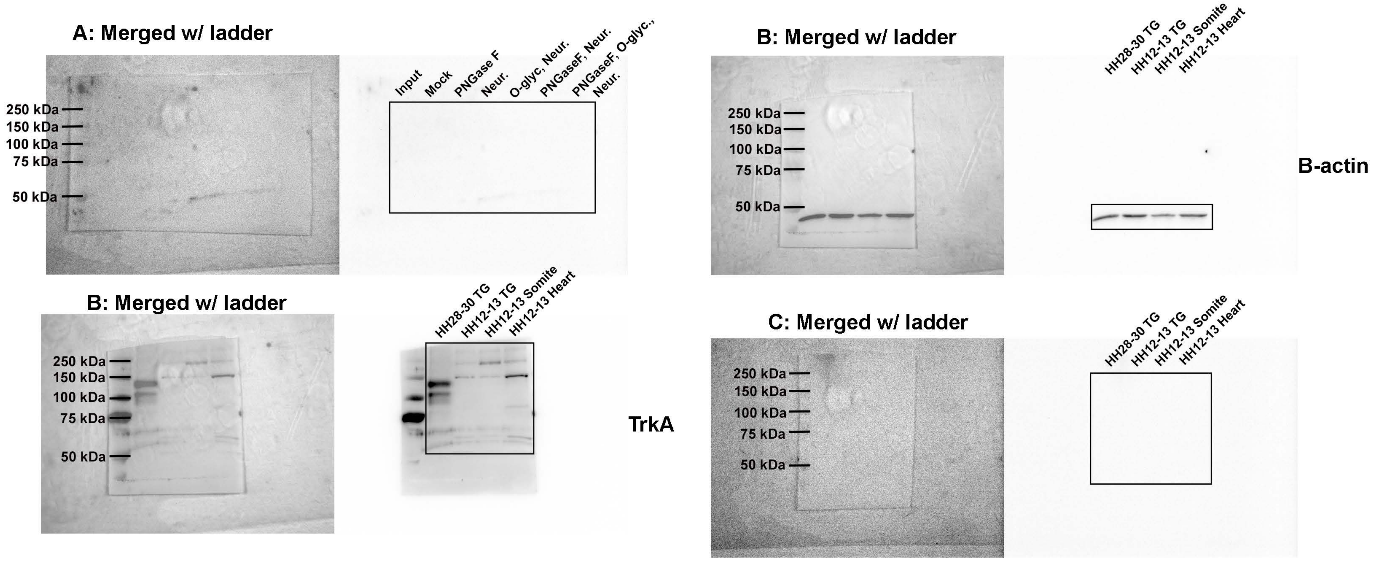

Fig. S3.

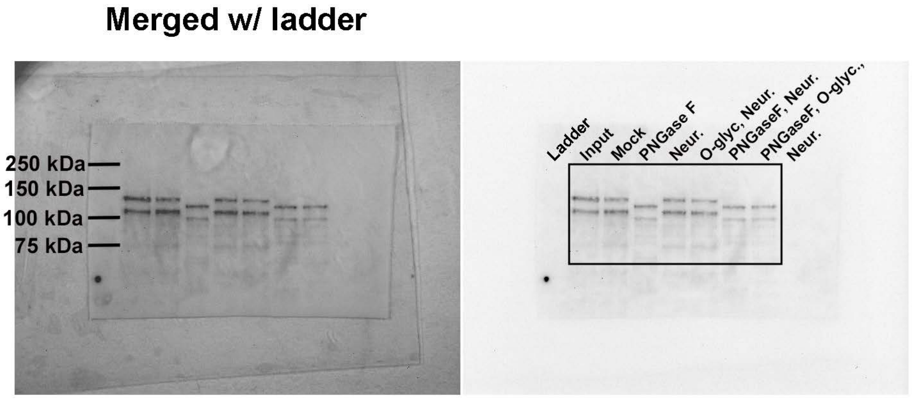

Fig. S6. Original western blots for main and supplemental figures. Black boxes indicate areas of blots featured in text.

Journal of Cell Science • Supplementary information
