# Supplementary material for: Multiple glycoforms of TrkA interact with N-cadherin during trigeminal ganglion neurodevelopment
Source: J Cell Sci. 2026 May 11;139(9):jcs264598. doi: 10.1242/jcs.264598 (PMC13245897; doi:10.1242/jcs.264598)
Supplement: Supplementary file 1 [file joces-139-264598_review_history.pdf]

Journal of Cell Science

Multiple glycoforms of TrkA interact with N-cadherin during trigeminal ganglion neurodevelopment

Caroline A. Halmi and Lisa A. Taneyhill
DOI: 10.1242/jcs.264598

Editor: Andrew Ewald

Review timeline

Original submission:	14 November 2025
Editorial decision:	12 December 2025
First revision received:	30 January 2026
Editorial decision:	12 February 2026
Second revision received:	17 March 2026
Accepted:	19 March 2026

Original submission

First decision letter

MS ID#: jcs.264598

MS TITLE: Multiple glycoforms of TrkA interact with N-cadherin during trigeminal ganglion neurodevelopment

AUTHORS: Lisa Anne Taneyhill; Caroline Halmi

ARTICLE TYPE: Research Article

Dear Dr Taneyhill,

We have now reached a decision on the above manuscript.

As you will see, Reviewer 1 has only minor suggestions and Reviewer 2 has identified multiple claims that need further validation and also suggests multiple additional lines of experiments that would strengthen the study. They suggest, however, that a revised version might prove acceptable, if you can address their concerns. I agree with Reviewer 2 that all of the suggested experiments are interesting and if you are in a position to complete them all that would be terrific. However, since time and reagents are often limited, I suggest below which ones are most important to the success of this manuscript, following the numbering in Major Concerns for Reviewer 2. (1) is important as it does remain possible that this antibody is detecting both TrkA and other proteins. (2) is useful though it needn't cover every single compartment mentioned. Higher magnification images would be useful. (3) is less important unless your lab is already highly adept at surface biotinylation assays; they are complex assays and subject to their own caveats and are more commonly the start of a new study than the finish of an existing one. (4) is reasonable at the level of testing for effects on outgrowth and survival. It is possible that one or more has toxic effects in these cultures. (5) can likely be handled by revisions to the text to ensure that you are precise about what has been shown in lysates and blots vs. what could only be shown in live cell colocalization / FRET / other experiments that directly measure interaction at the membrane in live cells. Please feel free to suggest a revision plan for my review if you would like further input. If you think that you can deal satisfactorily with the criticisms on revision, I would be pleased to see a revised manuscript. We would then return it to the reviewers.

© 2026. Published by The Company of Biologists under the terms of the Creative Commons Attribution License (https://creativecommons.org/licenses/by/4.0/).

Reviewer 1

Advance summary and potential significance to field

In this manuscript, Halmi and Taneyhill characterize the post-translational modifications (glycosylation) of NGF receptor, TrkA, in the chicken trigeminal ganglia and report its physical interaction with N-cadherin at the plasma membrane of trigeminal neurons. Although the work is largely descriptive, defining the glycosylation state of TrkA is an important first step for future studies to uncover the role and significance of these glycoforms for proper neurodevelopment. The work is well executed and presented. I have just a couple of comments and suggestions.

Comments for the author

Figure 2 - In cultured trigeminal ganglia the authors report a TrkA form at 223 kD (Fig 2E) that is not found in ganglia lysates (Fig 1). They speculate it could represent a TrkA form bound to NGF, however it is unclear as to why it would be present in one preparation and not the other.

The discussion is rather dense and lengthy and could be streamlined. The authors should also consider including a table summarizing the different TrkA glycoforms generated under various conditions, this would be a very useful guide for the reader.

Reviewer 2

Advance summary and potential significance to field

In this study, Halmi and Taneyhill address the in vivo glycosylation profile of TrkA receptors in the chick trigeminal ganglion. The authors show that TrkA receptors undergo N-linked glycosylation and incorporate sialic acid residues. Notably, they also report the presence of a subset of TrkA receptors bearing O-linked glycans. Through pharmacological perturbation of the secretory pathway in ganglia explants, they report that TrkA localization is altered and that specific post-translationally modified forms of the receptor become enriched. Using immunofluorescence and co-immunoprecipitation analyses, the authors suggest a potential interaction between TrkA and N-cadherin at both intracellular membranes and the plasma membrane within trigeminal ganglion neurons and their axons. Overall, the study addresses an interesting question about the glycosylation of TrkA receptors-compared to receptor phosphorylation, relatively few studies have addressed glycosylation modifications or their role in receptor localization and function, particularly in vivo. However, the study misses several key experiments necessary to fully support the conclusions, and the quality of the data can be improved.
Major concerns:
1. For the banding patterns attributed to TrkA in control conditions, it will be important to compare with a TrkA knockout condition (in the background of Bax deletion to prevent neuronal apoptosis) to address which bands are indeed differently modified versions of TrkA or arising from artifacts arising from antibody specificity and lysate preparation/immunoblotting. For example, the authors identify a 223 kD band as TrkA that shows up in some immunoblots, but not in others, despite its molecular weight differing from that typically observed in vivo and in other ex vivo preparations. This discrepancy is attributed to possible SDS-PAGE artifacts. To resolve the inconsistencies in banding patterns and strengthen their conclusions, it is recommended that the authors provide additional evidence that the bands are indeed modified forms of TrkA.

2. Overall, the authors should support their conclusions of sub-cellular localization of TrkA with available markers for distinct compartments, ER, Golgi, plasma membrane, endosomes etc. If antibodies compatible with labeling the relevant organelles are not available, the authors should consider using cytoskeletal markers (b-III-tubulin) to delineate neuronal morphology. This would provide essential structural context and improve the clarity of the images. In general, the authors could provide better quality images to show TrkA localization (Figure 2), TrkA and N-Cadherin co-localization (Figure 3). Additionally, the quality and interpretability of the imaging data would be improved by including insets with magnified views of axons and cell bodies. Further, quantitative

© 2026. Published by The Company of Biologists under the terms of the Creative Commons Attribution License (https://creativecommons.org/licenses/by/4.0/).

assessment of TrkA/N-cadherin colocalization, both at the cell surface and intracellularly, would further strengthen the rigor, consistency, and reproducibility of the findings.

3. The authors propose that TrkA does not reach the plasma membrane following disruption of the secretory pathway. Given that the antibody used recognizes an extracellular epitope, it is important to directly assess surface localization. To strengthen this conclusion, the authors should perform surface labeling of TrkA receptors in vitro. Alternatively, a surface biotinylation assay using membrane-impermeant biotin would allow for a more definitive evaluation of TrkA presence at the plasma membrane

4. Do the pharmacological agents (monensin, Brefeldin, tunicamycin) affect neurite outgrowth or neuronal survival? For example, the authors report that tunicamycin results in an overall reduction in TrkA levels. Is this because of detrimental effects on neurite growth/cell survival?

5. The authors speculate that the interaction between the neurotrophic receptor TrkA and the cell-adhesion molecule N-cadherin may play an important role in neurodevelopment. However, no experimental evidence is provided to support this hypothesis. To strengthen the manuscript and substantiate this proposed functional relationship, the authors should demonstrate that TrkA and N-cadherin indeed interact at the plasma membrane and assess relevant functional outcomes of disrupting N-cadherin presence, such as effects on TrkA receptor localization, or neurotrophic signaling (downstream signaling).

Minor:

1. It is not clear how the data presented in Figures 3J and 3K, and specially the percentage of each TrkA band, relate to the analysis of TrkA colocalization with N-cadherin. The connection between these datasets should be clarified.

2. The Methods section lists two different antibodies for TrkA immunoblotting. For clarity and reproducibility, the authors should specify in the figure legends which TrkA antibody was used for each blot presented in the figures.

First revision

<u>Author response to reviewers' comments</u>

Dear Andy:

We wish to thank you and the reviewers for your consideration and comments regarding our submitted manuscript entitled “Multiple glycoforms of TrkA interact with N-cadherin during trigeminal ganglion neurodevelopment” (MS ID#: jcs.264598). We are pleased that the reviewers liked the manuscript, and as requested by them and yourself, we have revised the manuscript and provided additional clarification according to their comments. Changes made to the manuscript text are highlighted in yellow.

Reviewer 1

1. The Reviewer writes “in cultured trigeminal ganglia the authors report a TrkA form at 223 kD (Fig. 2E) that is not found in ganglia lysates (Fig. 1). They speculate it could represent a TrkA form bound to NGF, however it is unclear as to why it would be present in one preparation and not the other.”

We thank the Reviewer for pointing this out and apologize for the lack of clarity in our text. Indeed, this TrkA form at 223 kD is present in multiple preparations of trigeminal ganglia lysate, both *in vivo* (Figures 1 and 4; Supplemental Figures 1 and 2) and in *ex vivo* cultures (Figures 2 and 3). Because our lysate is prepared from pooled trigeminal ganglia across three different stages (HH28-HH30), it is possible this 223 kD TrkA form is predominantly found in one stage versus the

© 2026. Published by The Company of Biologists under the terms of the Creative Commons Attribution License (https://creativecommons.org/licenses/by/4.0/).

others. As such, any variability in its presence is likely do to the skewing of embryo collection.

2. The Reviewer indicates that "the discussion is rather dense and lengthy and could be streamlined. The authors should also consider including a table summarizing the different TrkA glycoforms generated under various conditions, this would be a very useful guide for the reader."

We appreciate these suggestions by the Reviewer. To this end, we have streamlined the discussion section and have also generated two tables to summarize results obtained after *in vivo* enzymatic treatment and glycan enrichment.

Reviewer 2

1. *The Reviewer writes that it is "important to compare [the banding patterns attributed to TrkA in control conditions] with a TrkA knockout condition…to address which bands are indeed differently modified versions of TrkA or arising from artifacts from antibody specificity and lysate preparation/immunoblotting."*

We thank the Reviewer for raising this critical point to provide additional evidence that the bands are modified forms of TrkA. Unfortunately, it is not possible for us to generate a TrkA (or other) knockout in the chick embryo as is routinely done in a model like the mouse. While we recognize that this lack of genetic tractability is a limitation of our system, we have implemented a multipronged approach to address this concern as follows. First, we have performed immunoblotting on the lysates prepared after the various enzymatic treatments of trigeminal ganglia (TrkA-positive), but in the absence of the TrkA primary antibody (a "secondary antibody only" control). This is the "gold standard" experiment used in immunoblotting to demonstrate specificity of the primary antibody. In this experiment, we observed no bands, supporting that the bands we identified are in fact TrkA-immunoreactive (Supplemental Figure 2A). These bands are consistent with what has been reported in the literature (Julien et al., 2002), particularly after treatment with different enzymes (Watson et al., 1998).

Second, we conducted immunoblotting on tissues that were purportedly TrkA-negative based upon the absence of expression observed by *in situ* hybridization - HH12-HH13 forming heart and somite/lateral plate mesoderm (Cotrina et al., 2000). On this blot, we also included our standard HH28-HH30 dissected trigeminal ganglia as well as "forming" trigeminal ganglia from migratory neural crest cells and newly delaminated/ migrating placodal neuroblasts at HH12-HH13 since our RNA-sequencing data identified *TrkA* transcripts at HH13 (Leonard et al., 2025), and we were curious as to the banding pattern presented by TrkA protein in this stage group. Similar TrkA immunoreactive bands were observed in all dissected tissues at HH12-HH13; specifically, three higher molecular weight bands calculated to be 326, 246, and 161 kD, and a unique 101 kD band in the somite/lateral plate mesoderm sample (Supplemental Figure 2B). Upon further examination of the literature, we identified two reports that documented *TrkA* transcripts by RNA sequencing in the HH12-HH13 heart (Anderson et al., 2019) and HH12-HH13 somites/lateral mesoderm (Mok et al., 2024). Intriguingly, the 137 kD and 107 kD bands that are present in HH28-HH30 trigeminal ganglia were noticeably absent in these HH12-HH13 tissues that express *TrkA*. Taken together, these results suggest that the 137 kD and 107 kD forms of TrkA may be specific to mature neurons, at least those present in the trigeminal ganglion. In further support of this, a secondary antibody only control showed no bands (Supplemental Figure 2), once again confirming TrkA antibody specificity.

Third, we verified that the epitope of our TrkA antibody (the extracellular domain) is specific to chick TrkA by blasting it against the *Gallus gallus* proteome, with NT-3 Growth Factor Receptor (TrkC) being the next closest sequence. It shares only 39% similarity with the epitope, which, based on our experience using antibodies in chick and in cell lines, is not similar enough to permit immunoreactivity. We revisited our lectin binding assay data (Figure 1B), which also identified TrkA forms that were observed solely via immunoblotting and not with any glycan enrichment, and as seen in Jullien et al. (2002). In this experiment, we performed "bead only controls" (Figure 1 blot transparency), and TrkA was only enriched in the presence of the different lectin binders (ConA, WGA, PNA).

It is worth noting that the higher molecular weight forms of TrkA we observe are in keeping with what has been published previously (Watson et al., 1998), including TrkA bound to NGF (Hartman et al., 1992), but could also represent 1) TrkA "multimers" (i.e., dimers) that have not been completely denatured after addition of Laemmli buffer or boiling and/or have aggregated (see Shen and Maruyama (2011) for the presence of TrkA dimers even after denaturation and in

© 2026. Published by The Company of Biologists under the terms of the Creative Commons Attribution License (https://creativecommons.org/licenses/by/4.0/).

the absence of NGF; see Hartman et al. (1992) where TrkA bands at ~220 and ~300 kD were immunoprecipitated from PC12 cells, similar to the bands we observe in our immunoblotting experiments) and/or 2) more heavily glycosylated forms of TrkA that have yet to be "pruned," as seen for other glycosylated proteins/receptors (Scott et al., 2013; Xiang et al., 2013; Zhen et al., 2003). We hope the Reviewer finds these experiments acceptable given the limitations of our system, and thank the Reviewer again for this suggestion, as these new experiments have strengthened the manuscript.

2. The Reviewer indicates that "the authors should support their conclusions of sub-cellular localization of TrkA with available markers for distinct compartments. If antibodies [are not] compatible…the authors should consider using cytoskeletal markers [like] beta-III-tubulin [Tubb3] to delineate neuronal morphology." The authors should also supply "better quality images to show TrkA localization (Figure 2) [and] TrkA and N- cadherin co-localization (Figure 3)…[and] quantitative assessment of TrkA/N-cadherin colocalization…[to] further strengthen the rigor, consistency, and reproducibility of the findings."

We thank the Reviewer for providing this feedback. Prior to the submission of this manuscript, we attempted to visualize organelles by performing immunostaining on, or staining of, our *in vivo* tissue sections and *ex vivo* explant cultures for the ER (e.g., SERCA2, ER-ID stain kit), Golgi (e.g., GM130, GORASP2, Cytopainter stain kit), and endosomes (e.g., Rab5, Rab7, Rab11). Unfortunately, none of these antibodies or staining kits was specific for chick tissue and yielded inconclusive, uninterpretable results. To provide additional evidence of TrkA distribution in neurons both normally and after inhibitor treatment, we next followed the Reviewer's suggestion and carried out immunostaining for TrkA and Tubb3 to provide essential structural context. This experiment allowed us to visualize the neuronal cytoskeleton, and its data further support our conclusions regarding changes in TrkA localization and distribution to cellular organelles like the ER and Golgi after inhibitor treatment, in the absence of organelle staining.

To further address concerns of the Reviewer, we have obtained better quality low and high magnification images for Figures 2 and 3. We have also quantified TrkA and N-cadherin colocalization, finding that 9.2% of TrkA-positive puncta colocalize with N-cadherin-positive puncta *in vivo* compared to 8% *ex vivo* (see new Figure 3). We thank the Reviewer for these helpful suggestions and hope the new images and quantification have alleviated any concerns.

3. The Reviewer suggests that we perform "a surface biotinylation assay using membrane-impermeant biotin [to] allow for a more definitive evaluation of TrkA presence at the plasma membrane" to strengthen the notion that "TrkA does not reach the plasma membrane following disruption of the secretory pathway."

We appreciate this suggestion by the Reviewer. However, we feel that this experiment is beyond the scope of this current study, as we are not adept at performing such complex assays, which are also subject to their own caveats. In lieu of this, we have modified the manuscript text to remove any speculation about TrkA trafficking to the plasma membrane following disruption of the secretory pathway.

4. The Reviewer asks whether the "pharmacological agents (monensin, Brefeldin, tunicamycin) affect neurite outgrowth or neuronal survival" and thus potentially alter TrkA levels.

This is an excellent point raised by the Reviewer. To address this point, we first quantified the total TrkA signal from our immunoblots (Figure 2E) and found no statistically significant reduction in total TrkA protein levels overall (Figure 2H). These results suggest that the treatments are not causing the distribution of TrkA forms to change (Figure 2F-G). Next, we used Tubb3 immunoreactivity to evaluate any changes to axons in our *ex vivo* explant cultures (Supplemental Figure 4). While some differences were evident, we did not observe any glaring changes to axon outgrowth, likely because the trigeminal ganglia are cultured at a developmental stage where there are already many axons present, together with the short incubation time of our cultures (4 hours).

To further address the question of neuron health, we measured the area of the nuclei of TrkA/Tubb3 double-positive neurons from all of our higher magnification images, using this as a proxy for cell health. We subtracted two standard deviations from the mean nucleus area in the DMSO control to give us a number that would represent anything outside the "normal" range of

© 2026. Published by The Company of Biologists under the terms of the Creative Commons Attribution License (https://creativecommons.org/licenses/by/4.0/).

nuclear size in the control. Through this analysis, we found no statistical significance in nucleus size between the DMSO control and Monensin, and DMSO control and Brefeldin A. However, Tunicamycin treatment led to a reduction in nucleus size ($p = 0.013$, Table 3). These data strongly support that changes noted in TrkA forms after Monensin and Brefeldin A treatment are due to a disruption of the secretory pathway and not from cell death. Conversely, we cannot rule out effects of cellular stress and death as a contributing factor to the changes in TrkA forms observed after Tunicamycin treatment. This is perhaps not surprising due to the known role of Tunicamycin as an ER stressor that can promote cell death (Heifetz et al., 1979, Chang and Korolev, 1996, Wang et al., 2016). We thank the Reviewer for bringing this to our attention, as the inclusion of this information has strengthened the manuscript.

5. Given that "the authors speculate that the interaction between [TrkA and N-cadherin] may play an important role in neurodevelopment," the Reviewer would like to see evidence of TrkA and N-cadherin interaction at the cell surface of neurons, as speculated in the text, and an assessment of the "relevant functional outcomes of disrupting N-cadherin presence."

We thank the Reviewer for bringing these experiments to our attention. To address this, we have now modified the text in the Abstract, Introduction, and Conclusions to be more precise about what we have discovered in our immunoblotting experiments and have removed more speculative statements that would require additional experimentation to substantiate a proposed functional relationship. Such experiments will serve as the foundation of our future studies, including directly measuring TrkA and N-cadherin interactions on the membrane of living cells and designing expression constructs for use in FRET or GFP reconstitution assays. We appreciate this feedback from the Reviewer as it will help guide new experiments moving forward.

Minor comments:

1. The Reviewer writes that "it is not clear how the data presented in Figures 3J and 3K, and specially the percentage of each TrkA band, relate to the analysis of TrkA colocalization with N-cadherin. The connection between these datasets should be clarified."

We thank the Reviewer for pointing this out, as we realize this data are extraneous and do not relate to TrkA and N-cadherin colocalization. As such, we have removed Figures 3J and 3K from the manuscript.

2. The Reviewer indicates that the "Methods section lists two different antibodies for TrkA immunoblotting. For clarity and reproducibility, the authors should specify in the figure legends which TrkA antibody was used for each blot presented in the figures."

We appreciate this important point raised by the Reviewer and have now clarified which antibody was used in the legend of each figure.

We wish to express our thanks to you and the Reviewers for providing these highly constructive comments that have clarified the last remaining aspects of the manuscript. With these additional modifications, we believe that the manuscript is now acceptable for publication. We look forward to hearing from you soon and again thank you for handling our paper.

Second decision letter

MS ID#: jcs.264598R1

MS TITLE: Multiple glycoforms of TrkA interact with N-cadherin during trigeminal ganglion neurodevelopment

AUTHORS: Caroline A. Halmi; Lisa Anne Taneyhill

ARTICLE TYPE: Research Article

© 2026. Published by The Company of Biologists under the terms of the Creative Commons Attribution License (https://creativecommons.org/licenses/by/4.0/).

Dear Dr Taneyhill,

We have now reached a decision on the above manuscript.

As you will see, the reviewers gave favourable reports but reviewer 2 raised one specific question about the neuronal analysis. Please read and respond whether you think that their proposed reanalysis would usefully provide further information about the phenotype. If you disagree, please rebut their concern. I hope that you will be able to carry these out because I would like to be able to accept your paper.

Second revision

Author response to reviewers' comments

Dear Andy:

We wish to thank you and the reviewers for your consideration and comments regarding our resubmitted manuscript entitled “Multiple glycoforms of TrkA interact with N-cadherin during trigeminal ganglion neurodevelopment” (MS ID#: jcs.264598). We are pleased that the reviewers liked the revised manuscript, and as requested by them and yourself, we have revised the manuscript and provided additional clarification according to their comments. Changes made to the manuscript text are highlighted in yellow.

Reviewer 1

The Reviewer writes “the revisions have nicely addressed” their minor concerns.

We are pleased the Reviewer likes the revisions and that they find the manuscript acceptable for publication.

Reviewer 2

The Reviewer writes that “the authors have addressed the majority of my concerns.” However, “a quantitative assessment of neurite outgrowth or neuronal numbers would be a rigorous assessment of neuronal/axonal viability” after treatment with the different pharmacological agents.

We appreciate the Reviewer raising this critical point with respect to more robustly evaluating neurons after treatment with Monensin, Brefeldin A, and Tunicamycin. To address this, we have now quantified the mean axonal area in our explant cultures by examining Tubb3 signal intensity, as per Halmi et al., 2025. Our results indicate that there is no statistically significant difference in mean axonal area after treatment with any pharmacological agent compared to control. We thank the Reviewer for this suggestion, as this new data complement and support our measurements of nuclear size after treatment and have further strengthened the manuscript.

We wish to express our thanks to you and the Reviewers for providing these highly constructive comments that have clarified the last remaining aspects of the manuscript. With these additional modifications, we believe that the manuscript is now acceptable for publication. We look forward to hearing from you soon and again thank you for handling our paper.

Third decision letter

© 2026. Published by The Company of Biologists under the terms of the Creative Commons Attribution License (https://creativecommons.org/licenses/by/4.0/).

MS ID#: jcs.264598R2

MS Title: Multiple glycoforms of TrkA interact with N-cadherin during trigeminal ganglion neurodevelopment

Authors: Caroline A. Halmi; Lisa Anne Taneyhill

Article Type: Research Article

Dear Dr Taneyhill,

I am happy to tell you that your manuscript has been accepted for publication in Journal of Cell Science, pending standard publication integrity checks.

© 2026. Published by The Company of Biologists under the terms of the Creative Commons Attribution License (https://creativecommons.org/licenses/by/4.0/).
